# Identification of reference genes for circulating microRNA analysis in colorectal cancer

**DOI:** 10.1038/srep35611

**Published:** 2016-10-19

**Authors:** Yanqin Niu, Yike Wu, Jinyong Huang, Qing Li, Kang Kang, Junle Qu, Furong Li, Deming Gou

**Affiliations:** 1 Shenzhen Key Laboratory of Microbial Genetic Engineering, College of Life Sciences, Shenzhen University, Shenzhen, Guangdong, 518060, China; 2 Key Laboratory of Optoelectronic Devices and Systems of Ministry of Education and Guangdong Province, College of Optoelectronic Engineering Shenzhen University, Shenzhen, Guangdong, 518060, China; 3 Department of Biochemistry and Molecular Biology, School of Basic Medical Sciences, Shenzhen University Health Sciences Center, Shenzhen, Guangdong, 518060,China; 4 Clinic Medical Research Center, Shenzhen People’s Hospital, the Second Affiliated Hospital, Ji’nan University, Shenzhen, Guangdong, 518060, China

## Abstract

Quantitative real-time PCR (qPCR) is the most frequently used method for measuring expression levels of microRNAs (miRNAs), which is based on normalization to endogenous references. Although circulating miRNAs have been regarded as potential non-invasive biomarker of disease, no study has been performed so far on reference miRNAs for normalization in colorectal cancer. In this study we tried to identify optimal reference miRNAs for qPCR analysis across colorectal cancer patients and healthy individuals. 485 blood-derived miRNAs were profiled in serum sample pools of both colorectal cancer and healthy control. Seven candidate miRNAs chosen from profiling results as well as three previous reported reference miRNAs were validated using qPCR in 30 colorectal cancer patients and 30 healthy individuals, and thereafter analyzed by statistical algorithms BestKeeper, geNorm and NormFinder. Taken together, hsa-miR-93-5p, hsa-miR-25-3p and hsa-miR-106b-5p were recommended as a set of suitable reference genes. More interestingly, the three miRNAs validated from 485 miRNAs are derived from a single primary transcript, indicting the cluster may be highly conserved in colorectal cancer. However, all three miRNAs differed significantly between healthy individuals and non-small cell lung cancer or breast cancer patients and could not be used as reference genes in the two types of cancer.

MicroRNAs (miRNAs) are small noncoding RNAs of ∼22 nucleotides that regulate gene expression at the post-transcriptional level^1^,^2^,^3^. Myriad reports established that miRNAs are involved in cell differentiation, proliferation, and apoptosis and are implicated in many types of disease^4^,^5^,^6^,^7^,^8^. It was shown that miRNA expression profiles differ between healthy and diseased tissue^9^,^10^,^11^. Meanwhile, blood have a cancer-associated miRNA signature, and circulating miRNA profiles correlate with patient diagnosis, prognosis and responses to treatment^12^,^13^,^14^,^15^. Thus, miRNAs in plasma/serum have shown promise as biomarkers of disease.

Because of high sensitivity, specificity and low template requirements, quantitative real-time PCR (qPCR) currently is most frequently used for measuring the expression of miRNAs^16^,^17^,^18^,^19^. The accuracy of qPCR-based miRNA expression profiling depends on normalization by reference genes. Moreover, release 21.0 of the miRBase database has cataloged 4552 human miRNAs (http://www.mirbase.org/index.shtml); over 30,000 associated literature references had been listed in the PubMed database (accessed on 31 August 2016). However, multiple results have so far been inconsistent about their relative performances and populations. Based on above, the optimal selection of reference genes to be used for normalization is critical for miRNA expression studies.

Colorectal cancer is the third most common cancer and the fourth leading cause of cancer-related deaths in the world^20^. At an early stage of development, it is curable; however, most patients have no phenotypic symptoms at this time^19^. Therefore, circulating miRNAs as potential predictive biomarkers are of great importance. Despite increasing miRNA expression studies has been reported in colorectal cancer, as far as we are aware, there is no current consensus on reference for qPCR analysis of circulating miRNAs in colorectal cancer.

In this article, we aim to develop miRNA profiling data normalization approach based on the selection from large scale of candidate miRNAs in colorectal cancer. First, 485 human miRNAs were selected and subsequently quantified with a sensitive qPCR-based assay, the S-Poly(T) Plus assay^21^, of which, 372 miRNAs that could be detected in human serum or plasma were obtained from QIAGEN website (http://www.sabiosciences.com/genetable.php?pcatn=MIHS-3106Z); 113 other blood-derived miRNAs were extracted from literatures with the key words “microRNA/miRNA”, “serum/plasma/blood” in combination with “human/hsa-”. Then, BestKeeper, geNorm and NormFinder algorithms were applied to identify the most stable reference genes. Finally, the recommended normalizer(s) was (were) validated in colorectal cancer as well as non-small cell lung cancer (NSCLC) and breast cancer.

## Results

### Selection of candidate reference miRNAs from profiling

**Figure 1:**
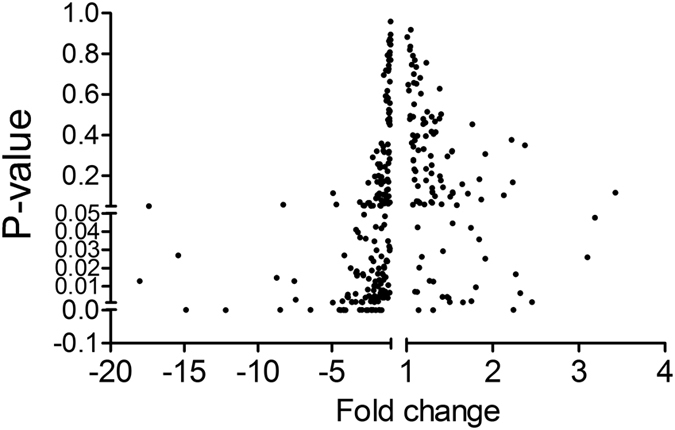
Volcano plot illustrates 485 miRNAs differentially expressed between colorectal cancer patients and healthy individuals.

To identify suitable reference genes across the healthy individuals and colorectal cancer patients, we have developed miRNA profiling data normalization approach based on the selection from large scale of candidate miRNAs. 485 miRNAs (Supplemental Table 1) were assessed with qPCR approach and were depicted in the volcano plot as in Fig. 1. Seven out of 485 putative reference miRNAs (hsa-miR-320d, hsa-miR-25-3p, hsa-miR-92b-3p, hsa-miR-106b-5p, hsa-miR-10b-5p, hsa-miR-107 and hsa-miR-574-5p) were selected based on the following criteria: (a) the Ct values of all miRNA were <32; (b) the fold change between the healthy populations and colorectal cancer patients was lower than 1.1; (c) no significant differences were existed between the healthy and diseased groups (P > 0.05). In addition, three candidates (hsa-miR-101-3p, hsa-miR-16-5p, and hsa-miR-93-5p) were also chosen as potential reference miRNAs according to literatures^22^,^23^,^24^,^25^,^26^. For the three miRNAs, we also used miRNA profiling data in this study to make sure consistent results. Taking together, ten putative reference miRNAs were subjected to further analyses (Table 1).

### Candidate miRNA ranking

**Figure 2:**
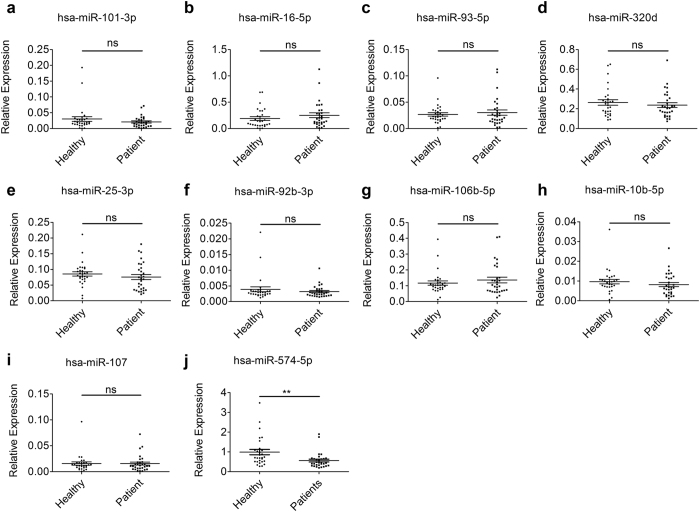
Ten candidate reference miRNAs in the serum between colorectal cancer patients (n = 30) and healthy individuals (n = 30). miRNA levels were normalized to spiked-in cel-miR-54-5p and represented in scatter plots. Data are shown as means ± SE, ns = not significant; **P < 0.01.

For confirmation, ten reference candidates selected were separately measured by qPCR in 30 healthy individuals and 30 colorectal cancer patients (randomly chosen from 86 healthy individuals and 66 colorectal cancer patients). The expression levels of all miRNAs showed no significant difference between the healthy and diseased groups, except hsa-miR-574-5p (P < 0.01) (Fig. 2). Therefore, we picked the left nine putative reference miRNAs and reassess their potential contributions as normalizers following the instructions of the BestKeeper, geNorm and NormFinder.

BestKeeper was first used to analyze the expression variability of each miRNA by calculating the Ct set standard deviation (SD). Genes with SD greater than 1 were assumed to be in consistent. This application ranked hsa-miR-320d, hsa-miR-92b-3p, hsa-miR-25-3p and hsa-miR-106b-5p as the least variable genes (SD values are 0.498, 0.748, 0.842 and 0.966, respectively), suggesting these four candidates could be used as reference genes in miRNA analysis of colorectal cancer (Table 2).

**Figure 3:**
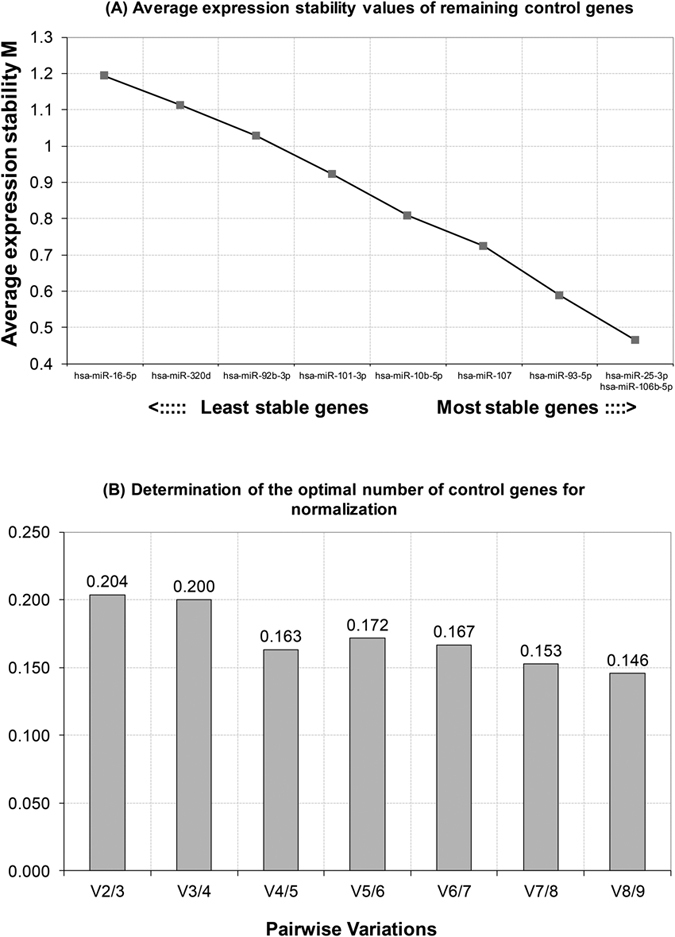
geNorm analysis of qPCR-based candidate reference genes. (**A**) Average expression stability values of remaining control genes. M was excluded in a stepwise manner until the two most stable genes remained: hsa-miR-25-3p and hsa-miR-106-5p. (**B**) Determination of the optimal number of control genes for normalization. The number of reference genes is considered optimal when the V value reached the lowest, at which point it is unnecessary to include additional genes in the normalization strategy.

Based on fold change data (see the statistical method in details), geNorm generates a stability value M using the average pair wise variation between all tested genes, accompanied by stepwise exclusion of the least stable gene until the two most stable genes were left (Fig. 3A). Genes with the highest M value have the least stable expression, whereas the genes with the lowest M value have the most sable expression. In this study, hsa-miR-106b-5p, hsa-miR-93-5p and hsa-miR-25-3p showed the lowest M values, indicating with the most stable expression (Table 1). In addition, geNorm calculates a normalization factor (V_NF_ value), which is a criterion for the optimal number reference genes. V_NF_ value of 1.5 was set as a cutoff for proper normalization. When this threshold achieved, it is not necessary to include any additional reference genes^27^. In our data sets, only V_8/9_ was less than 0.15, indicating the nine miRNAs combination as the best reference normalization factor (Fig. 3B). It is not so convenient for this combined reference set as a normalizer; therefore, this threshold should not be viewed too strictly.

Similar to geNorm, NormFinder evaluated most stable genes with the lowest M value. NormFinder also showed that hsa-miR-106b-5p was the most stable candidate reference gene with M value 0.228, followed by hsa-miR-25-3p and hsa-miR-93-5p with an M value of 0.269 and 0.283, respectively (Table 2).

Although displaying differently, the results provided by BestKeeper, geNorm and Normfinder had overlaps. BestKeeper indicated that hsa-miR-320d, hsa-miR-92b-3p, hsa-miR-25-3p and hsa-miR-106b-5p could be used as reference miRNAs; both geNorm and NornFinder recommended hsa-miR-106b-5p, hsa-miR-25-3p and hsa-miR-93-5p as top three references. Overall, hsa-miR-106b-5p and hsa-miR-25-3p were recommended as suitable reference miRNAs by three algorithms. hsa-miR-93-5p ranked second when it was normalized by geNorm and NormFinder, and had been reported as validated reference miRNA^23^,^24^,^25^. We therefore included hsa-miR-93-5p as another reference gene (Tables 1 and 2). Comparing with a single reference gene, the combination of more than one normalizer may increase the accuracy of quantification in qPCR study. We therefore propose that the set of miRNAs including hsa-miR-106b-5p, hsa-miR-25-3p and hsa-miR-93-5p could be used as reference genes as combination or separately in colorectal cancer. Then, there is no need for a spike-in reference, because RNA extraction efficiency will not have influence on the miRNA expression pattern when endogenous gene(s) was(were) used as normalizer(s).

To further demonstrate the applicability of the three reference miRNAs, another set of samples from a different hospital (Peking University Shenzhen Hospital) were collected. Sera were obtained from 30 healthy individuals and 30 colorectal cancer patients. The expression levels of hsa-miR-106b-5p, hsa-miR-25-3p and hsa-miR-93-5p still showed no significant difference between the healthy and diseased groups (Supplemental Fig. 1). Besides, the S-Poly(T) Plus method, was revaluated by comparing with commercial TaqMan microRNA assay kit (Applied Biosystems). It turned out that the S-Poly(T) Plus assay showed a 2.6~263-fold increase in sensitivity (Supplemental Fig. 2), indicating that the miRNA measurement method used in this study are reliable.

### Assessment of validated reference miRNAs in colorectal cancer

**Figure 4:**
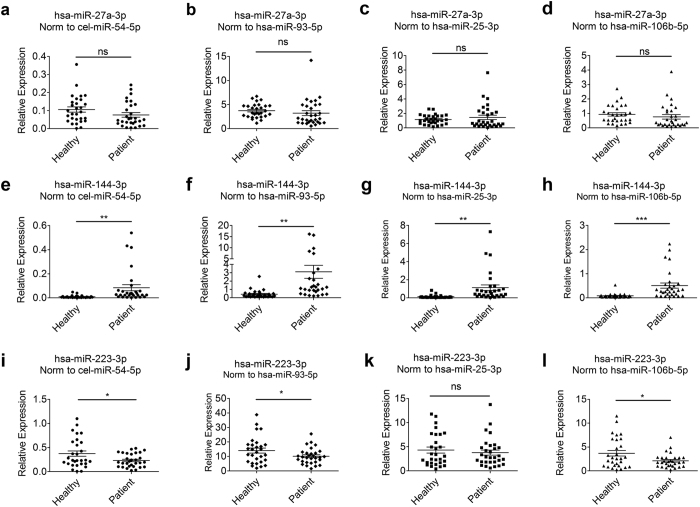
Expression levels of hsa-miR-27a-3p, hsa-miR-144-3p and hsa-miR-223-3p in colorectal cancer and healthy control. miRNAs were quantified in the plasma of 30 colorectal cancer patients and 30 healthy individuals. miRNA levels were normalized to cel-miR-54-5p (dot), hsa-miR-93-5p (diamond), hsa-miR-25-3p (square) and hsa-miR-106b-5p (triangle) respectively. Data are shown as means ± SE, ns = not significant, *P < 0.05, **P < 0.01 and ***P < 0.001.

To test the effect of the three reference miRNAs (hsa-miR-106b-5p, hsa-miR-25-3p and hsa-miR-93-5p) on the accuracy of qPCR results, we selected hsa-miR-27a-3p, hsa-miR-144-3p and hsa-miR-223-3p as target miRNAs, because of their potential prognostic value in colorectal cancer^28^,^29^,^30^. The three miRNAs were validated in each of the serum using another different set of samples, including 30 colorectal cancer patients and 30 healthy subjects individually. Spiked-in cel-miR-54-5p, hsa-miR-93-5p, hsa-miR-25-3p and hsa-miR-106b-5p were used as normalizer separately. We found that there was no significant difference for hsa-miR-27a-3p in colorectal cancer patient serum compared to the healthy controls when data were normalized to cel-miR-54-5p (P = 0.1253), hsa-miR-93-5p (P = 0.3755), hsa-miR-25-3p (P = 0.3711) and hsa-miR-106b-5p (P = 0.3990), respectively; hsa-miR-144-3p was significantly up-regulated in colorectal cancer patient group when data were normalized to the four normalizers but the tendency was more significant when hsa-miR-106b-5p was used as reference (P < 0.01, P < 0.01, P < 0.01 and P < 0.001, respectively); for hsa-miR-223-3p, the expression levels differed significantly when cel-miR-54-5p (P < 0.05), hsa-miR-93-5p (P < 0.05) and hsa-miR-106b-5p (P < 0.05) were served as normalizers respectively, but did not differ significantly when data were normalized to hsa-miR-25-3p (P = 0.5226) (Fig. 4).

### Revalidated reference miRNAs in non-small cell lung cancer and breast cancer

**Figure 5:**
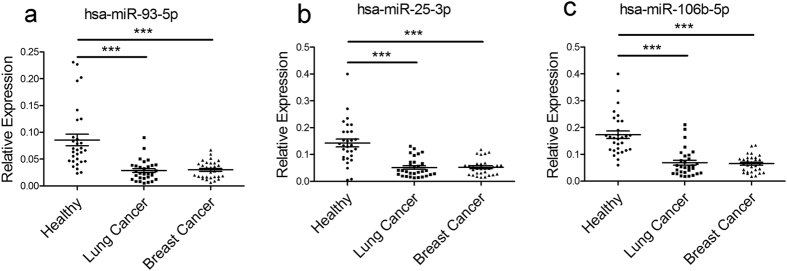
Expression levels of hsa-miR-93-5p, hsa-miR-25-3p and hsa-miR-106b-5p in non-small cell lung cancer and breast cancer. miRNAs were quantified in the plasma of 30 non-small cell lung cancer patients, 30 breast cancer patients and 30 healthy individuals. miRNA levels were normalized to spiked-in cel-miR-54-5p and represented in scatter plots. Data are shown as means ± SE, **P < 0.01, ***P < 0.001.

It was also tested whether one or a set of miRNAs could be used as universal reference genes for other types of cancer. Expression patterns of the top three reference miRNAs of colorectal cancer (hsa-miR-93-5p, hsa-miR-25-3p and hsa-miR-106b-5p) were determined in 30 healthy donors, 30 non-small cell lung cancer patients and 30 breast cancer patients, respectively. miRNA levels were normalized to spiked-in cel-miR-54-5p. The results showed that all three miRNAs differed significantly between healthy controls and non-small cell lung cancer patients or healthy controls and breast cancer patients, indicating that hsa-miR-93-5p, hsa-miR-25-3p and hsa-miR-106b-5p might be colorectal cancer specific as reference genes (Fig. 5). Moreover, it had been proved that other six candidate reference miRNAs (hsa-miR-107, hsa-miR-10b-5p, hsa-miR-92b-3p, hsa-miR-101-3p, hsa-miR-320d, and hsa-miR-574-5p) were not suitable as reference miRNAs for lung cancer or breast cancer (Supplemental Fig. 3). miR-16-5p was abandoned because there was signal in no-reverse transcriptase control in some breast cancer samples.

## Discussion

Since the mature miRNAs were first amplified and quantified by RT-PCR^31^, qPCR has been the most frequently used approach in miRNA expression studies. The accuracy of qPCR depends on suitable normalization of data inasmuch as inappropriate use of reference genes can significantly alter the results of target miRNA quantification. Numerous RNA species, including rRNA, snRNA, and miRNAs had been used as reference genes so far^34^,^35^,^36^. However, it has some problem of rRNA and snRNAs in serum or plasma in aspects of the abundance and stability.

In our study, we combined multiple strategies for identifying appropriate reference miRNAs. First, candidate reference genes were selected with qPCR approach from genome-wide serum miRNA expression profile (see the methods). To our knowledge, such assessment and validation of reference miRNAs for colorectal cancer studies has not be reported. Comparing to those reference genes identified by microarray or extracted from pervious literature, the accuracy and comparability of miRNA expression level data have been dramatically improved. Then, non-human miRNA cel-miR-54-5p from *Caenorhabditis elegans* was used as spiked-in in terms of less technical variation and more accurate identification of biological changes. Excluding technical variation, true reference genes should be stably expressed in the serum mixture pool and a larger cohort of colorectal cancer and healthy individuals. Therefore, after all miRNAs were profiled, putative reference miRNAs were validated in each sample; and candidate reference miRNAs with the least differences were confirmed and analyzed in BestKeeper, geNorm and NormFinder. Finally, hsa-miR-25-3p, hsa-miR-93-5p and hsa-miR-106b-5p were identified from 485 miRNAs as best set of reference genes in colorectal cancer.

Normalization strategies and candidate reference hsa-miR-106b-5p, hsa-miR-25-3p and hsa-miR-93-5p were validated by analyzing the expression of target miRNAs, hsa-miR-27a-3p, hsa-miR-144-3p and hsa-miR-223-3p. No matter the spiked-in cel-miR-54-5p, or hsa-miR-25-3p, hsa-miR-93-5p and hsa-miR-106b-5p were used as normalizers, there was no significant difference of the expression of hsa-miR-27a-3p between the colorectal cancer patients and healthy donors. This indicated that hsa-miR-25-3p, hsa-miR-93-5p, hsa-miR-106b-5p and spiked-in cel-miR-54-5p were all suitable reference genes in the serum between colorectal cancer patients and healthy controls. Moreover, as endogenous normalizers, hsa-miR-25-3p, hsa-miR-93-5p and hsa-miR-106b-5p may be more effective than exogenous spiked-in normalizer with more convinience and less error in performance.

hsa-miR-144-3p has been reported as potential biomarker in colorectal cancer and its forced expression was associated with malignant potential and prognosis^30^,^37^. In our study, hsa-miR-144-3p was significantly up-regulated in colorectal cancer patients comparing to healthy donors when cel-miR-54-5p, hsa-miR-93-5p, hsa-miR-25-3p and hsa-miR-106b-5p were served as normalizers. It confirms the suitability of hsa-miR-93-5p, hsa-miR-25-3p and hsa-miR-106b-5p to be used as optimal reference genes in the quantification. On the other hand, the difference was more significant when hsa-miR-144-3p were normalized to hsa-miR-106b-5p (Fig. 4). These results indicated that normalization to systematically selected reference genes could detect a miRNA even with smaller differences, and that hsa-miR-106b-5p could be somewhat better than hsa-miR-25-3p and hsa-miR-93-5p as a normalizer.

hsa-miR-223 has been frequently observed to be aberrantly expressed in varieties of tumor^34^,^38^,^39^, but there was no consistent results about the expression levels in colorectal cancer. Overexpression of hsa-miR-223 had been reported in colorectal adenocarcinomas^40^; however, evidences revealed that there was no significant difference between colorectal cancer patients and controls for hsa-miR-223 in plasma^28^ or tissue^41^. Regardless of different in samples, these results have drawn particular attention to the effect of references in normalizing the results, and demonstrated the urgent need for identification of suitable reference genes to produce reliable data. In our results, the expression level of hsa-miR-223-3p varied differently when cel-miR-54-5p, hsa-miR-93-5p and hsa-miR-106b-5p were used as normalizer but not when data normalized to hsa-miR-25-3p. Considering that it was consistent when hsa-miR-106b-5p, cel-miR-54-5p and hsa-miR-93-5p were used as normalizers, we therefore believed that it hsa-miR-223-3p was significant down-regulated tendencybetween the colorectal cancer patient and healthy control in our study.

This study was the first systematic investigation to identify appropriate reference miRNAs in colorectal cancer. We have identified hsa-miR-106b-5p, hsa-miR-93-5p and hsa-miR-25-3p as a set of best reference genes for qPCR normalization analysis in colorectal cancer. To our surprise, these three candidate references validated from 485 miRNAs were located in a single primary transcript on chromosome 7, indicating the function of miR-106b-25 (miR-106b/miR-93/miR-25) cluster may be highly conserved in colorectal cancer. Meanwhile, it also proved that the strategies we used in the identification of reference miRNAs in clinical serum sample between controls and diseased group are reliable.

We also evaluated the expression pattern of hsa-miR-25-3p, hsa-miR-93-5p and hsa-miR-106b-5p in non-small cell lung cancer and breast cancer. However, these miRNAs were significantly down-regulated in cancer patients and hence could not be used as references. Whether they could be used as reference genes for other cancers remains to be determined.

## Methods

### Serum collection

Human sera were collected from donors in Shenzhen People’s Hospital (Shenzhen, China) including 86 healthy donors, 66 patients with colorectal cancer, 30 patients with non-small cell lung cancer and 30 patients with breast cancer. Unless stated, samples used in this study were from this hospital. Another set of serum samples were collected from 30 healthy individuals and 30 colorectal cancer patients who came from Peking University Shenzhen Hospital (Shenzhen, China). The information of all donors was detailed in Table 3. The blood samples were kept at room temperature for 1 h and then centrifuged at 3,000 × g for 10 min at 4 °C. The sera were then stored at −80 °C. All procedures were carried out according to the approved guidelines. Both the two Institutional Ethics Committees at the Shenzhen People’s Hospital (Shenzhen, China) and Peking University Shenzhen Hospital (Shenzhen, China) approved the experiments, and written informed consent was obtained from all subjects.

### RNA extraction

Total RNA was isolated using our developed serum/plasma (S/P) miRsol method as previous described^21^. Of note, 0.1 pM spiked-in *Caenorhabditis elegans* cel-miR-54-5p was used for normalization and determination of technical variation in RNA extraction.

### Polyadenylation, reverse transcription and Real-time PCR

The polyadenylation, reverse transcription and Real-time PCR procedure were conducted exactly according to S-Poly(T) Plus protocol^21^. To profile miRNAs effectively, every 7 out of 485 miRNAs as well as spiked-in cel-miR-54 were grouped together for the one-step reaction of polyadenylation and reverse transcription, simultaneously. miRNAs with identical forward primers or with more than five base-pairings between the forward primer and RT primers have been avoided in the same group. All sequences, primers and probes were listed in the Supplemental Table 1. Besides, the S-Poly(T) Plus assay was evaluated by comparing with TaqMan microRNA assay kit (Applied Biosystems) according to the manufacturer’s instructions. No-template control (NTC) and no-reverse transcriptase control (-RT) were conducted simultaneously.

Real-time PCR was performed in 96-well plates by using ABI StepOne Plus thermal cycler. Each PCR reaction was carried out in duplicate. The miRNA expression level was normalized to spiked-in cel-miR-54-5p. miRNAs with cycle threshold (Ct) value less than 35 in the panel were included in data analysis.

### Selection and validation of reference miRNAs

A three-step test was designed to select and validate reference miRNAs for colorectal cancer. In the initial screening, we pooled serum samples of 86 healthy subjects and 66 patients with colorectal cancer respectively. 485 miRNAs from the two pools were detected using the S-Poly(T) Plus method. Then, miRNAs without significant difference between colorectal cancer group and healthy group (fold change < 1.1 and *P* > 0.05) were selected for validation in each individual of 30 colorectal cancer patients and 30 healthy controls (randomly chosen from 86 colorectal cancer patients and 66 healthy donors). miRNA(s) stably expressed in the serum mixture pool and individuals was (were) selected as most suitable candidate reference(s) in colorectal cancer. Ultimately, we selected hsa-miR-27a-3p, hsa-miR-144-3p and hsa-miR-223-3p as target miRNAs to test the effect of the candidate reference(s) on the accuracy of qPCR results.

Moreover, to find out if the selected reference(s) also stably expressed in other diseases, it (they) was (were) validated in each individual of 30 non-small cell lung cancer and 30 breast cancer patients. Fold change of miRNAs were calculated using the 2^−ΔΔCt^ method and spiked-in cel-miR-54-5p was used as a normalization control.

### Statistics

Statistical analysis was carried out using the GraphPad Prism 5 using two-tailed Student’s test. Data were present as means ± SE (standard error). P value of less than 0.05 was considered statistically significant. Mean values were obtained from SPSS software, version19.0 (SPSS Inc., Chicago, IL, USA). The stability of the candidate reference genes was evaluated by the computer program BestKeeper^42^, geNorm^27^ and NormFinder^43^. In the BestKeeper software, data observations are in the form of raw threshold cycles, while fold change were used as input data in both geNorm and NormFinder.

## Additional Information

**How to cite this article**: Niu, Y. *et al*. Identification of reference genes for circulating microRNA analysis in colorectal cancer. *Sci. Rep.* **6**, 35611; doi: 10.1038/srep35611 (2016).

## Supplementary Material

Supplementary Figure 1

Supplementary Figure 2

Supplementary Figure 3

Supplementary Table 1

## Figures and Tables

**Table 1:** Relative quantities of candidate reference miRNAs in gnome-wild serum profile.

miRNA	Ct (Healthy) (Mean ± SE)	Ct (Patient) (Mean ± SE)	Fold change	P value
hsa-miR-101-3p	27.70 ± 0.02	28.09 ± 0.06	−1.309	0.024
hsa-miR-16-5p	25.88 ± 0.02	25.64 ± 0.04	1.180	0.026
hsa-miR-93-5p	30.31 ± 0.04	31.22 ± 0.01	−1.873	0.002
hsa-miR-320d	28.76 ± 0.01	28.85 ± 0.01	−1.069	0.051
hsa-miR-25-3p	28.24 ± 0.01	28.34 ± 0.03	−1.067	0.069
hsa-miR-92b-3p	31.91 ± 0.20	31.96 ± 0.20	−1.030	0.895
hsa-miR-106b-5p	28.69 ± 0.04	28.65 ± 0.05	1.028	0.619
hsa-miR-10b-5p	32.05 ± 0.08	31.99 ± 0.01	1.045	0.495
hsa-miR-107	31.16 ± 0.03	31.07 ± 0.07	1.061	0.362
hsa-miR-574-5p	24.85 ± 0.22	24.77 ± 0.09	1.062	0.747

Hsa-miR-101-3p, hsa-miR-16-5p and hsa-miR-93-5p have been reported as normalizers in literatures; seven additional putative miRNAs (hsa-miR-320d, hsa-miR-25-3p, hsa-miR-92b-3p, hsa-miR-106b-5p, hsa-miR-10b-5p, hsa-miR-107 and hsa-miR-574-5p) were extracted with standards: fold change < 1.1 and P value > 0.05. Data of ten miRNA was from profiling data set in this study to make sure consistent results. All the Ct value was less than 32.

**Table 2:** Stability values and ranking of candidate reference genes were determined by BestKeeper, Normfinder and geNorm.

miRNA	BestKeeper		geNorm		NormFinder	
	std dev [± CP]	Rank	Stability value (M)	Rank	Stability value	Rank
hsa-miR-101-3p	1.413	8	1.334	9	0.730	6
hsa-miR-16-5p	1.820	9	1.474	8	0.878	8
hsa-miR-93-5p	1.297	6	0.960	2	0.269	2
hsa-miR-320d	0.498	1	1.471	8	0.906	9
hsa-miR-25-3p	0.842	3	0.977	3	0.283	3
hsa-miR-92b-3p	0.748	2	1.327	6	0.757	7
hsa-miR-106b-5p	0.966	4	0.944	1	0.228	1
hsa-miR-10b-5p	1.267	5	1.135	5	0.498	5
hsa-miR-107	1.394	7	1.113	4	0.420	4

The stability is indicated by standard deviation of crossing point (CP) values in Bestkeeper, a lower stability value (M) in geNorm and stability value in Normfinder.

**Table 3:** Characteristics of patients and healthy controls enrolled in this study.

Variables	Healthy	Colorectal Cancer	Non-small Cell Lung Cancer	Breast Cancer	Healthy (Peking University Shenzhen Hospital)	Colorectal cancer (Peking University Shenzhen Hospital)
Number	86	66	30	30	30	30
Gender						
Male	43	41	16	1	17	17
Female	43	25	14	29	13	13
Age						
Range	20~82	35~81	39~76	33~73	31~77	39~79
Mean ± SE	50.63 ± 1.89	57.91 ± 1.44	59.10 ± 1.72	48.87 ± 1.74	45.68 ± 1.98	48.93 ± 1.52
TNM stage						
0	—	3	0	0	—	1
I	—	7	8	6	—	5
II	—	21	6	12	—	12
III	—	22	8	9	—	8
IV	—	13	8	3	—	4

Unless marked, samples used in this study were from Shenzhen People’s Hospital (Shenzhen, China).
