# Supplementary material for: Identification of reference genes for circulating microRNA analysis in colorectal cancer
Source: Sci Rep. 2016 Oct 19;6:35611. doi: 10.1038/srep35611 (PMC5069661; doi:10.1038/srep35611)

Identification of reference genes for circulating microRNA analysis in colorectal cancer

Yanqin Niu[1, 2*], Yike Wu[1*], Jinyong Huang[1], Qing Li[1], Kang Kang[3], Junle Qu[2], Furong Li[4#] and Deming Gou[1#]

Supplemental figure 1 Expression levels of three reference miRNAs in serum samples from Peking University Shenzhen Hospital (Shenzhen, China). hsa-miR-93-5p, hsa-miR-25-3p and hsa-miR-106b-5p were revalidated in other 30 colorectal cancer patients and 30 healthy individuals. miRNA levels were normalized to spiked-in cel-miR-54-5p and represented in scatter plots. Data are shown as means ±SE, ns= not significant.

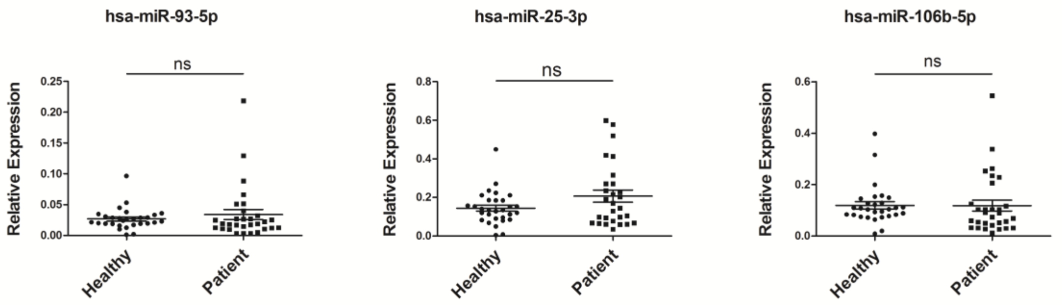

Supplement: Supplementary Figure 1 [file srep35611-s1.pdf]
