# Supplementary material for: Identification of reference genes for circulating microRNA analysis in colorectal cancer
Source: Sci Rep. 2016 Oct 19;6:35611. doi: 10.1038/srep35611 (PMC5069661; doi:10.1038/srep35611)
Supplement: Supplementary Figure 2 [file srep35611-s2.pdf]

Identification of reference genes for circulating microRNA analysis in colorectal cancer

Yanqin Niu[1, 2*], Yike Wu[1*], Jinyong Huang[1], Qing Li[1], Kang Kang[3], Junle Qu[2], Furong Li[4#] and Deming Gou[1#]

Supplemental Figure 2 Comparison of the sensitivity of two different miRNA qPCR assays. Stem-loop method was carried out with the TaqMan microRNA assay kit (Applied Biosystems) according to the manufacturer's instructions; S-Poly(T) Plus method was performed as previous described in Niu et al., 2015[1-2]. hsa-miR-140-5p, hsa-miR-124a-3p, hsa-miR-16-5p, hsa-miR-93-5p, hsa-miR-25-3p and miR-106b-5p were validated using the two methods, respectively. The S-Poly(T) Plus assay showed a 2.6～263-fold increase in sensitivity (1.8-8 threshold cycles difference).

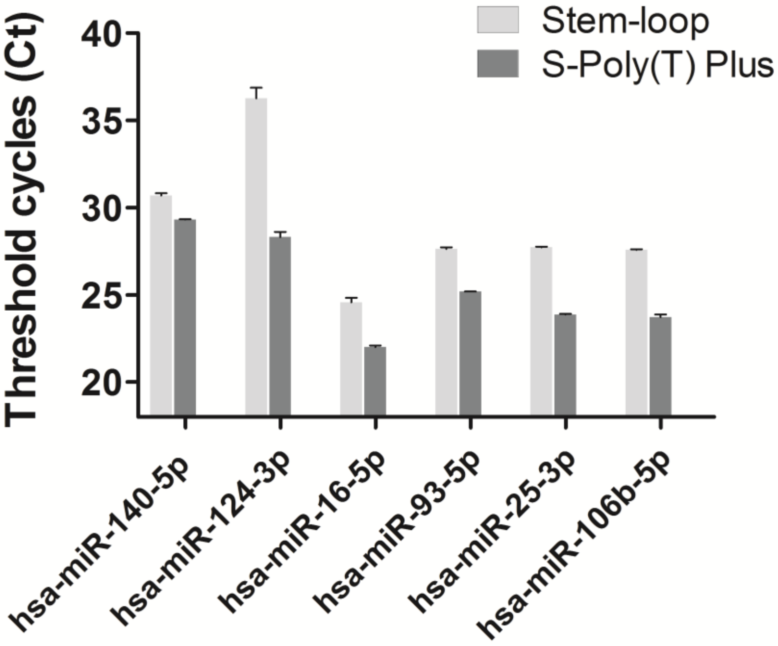

1. Niu Y, Zhang L, Qiu H, Wu Y, Wang Z, Zai Y, et al. An improved method for detecting circulating microRNAs with S-Poly (T) Plus real-time PCR. Scientific reports. 2015;5.

2. Kang K, Zhang X, Liu H, Wang Z, Zhong J, Huang Z, et al. A novel real-time PCR assay of microRNAs using S-Poly (T), a specific oligo (dT) reverse transcription primer with excellent sensitivity and specificity. 2012.
