# Supplementary material for: Identification of reference genes for circulating microRNA analysis in colorectal cancer
Source: Sci Rep. 2016 Oct 19;6:35611. doi: 10.1038/srep35611 (PMC5069661; doi:10.1038/srep35611)

Identification of reference genes for circulating microRNA analysis in colorectal cancer

Yanqin Niu[1, 2*], Yike Wu[1*], Jinyong Huang[1], Qing Li[1], Kang Kang[3], Junle Qu[2], Furong Li[4#] and Deming Gou[1#]

Supplemental figure 3 Expression levels of six candidate references of colorectal cancer in non-small cell lung cancer and breast cancer. miRNAs were validated in serum of 30 healthy donors, 30 non-small cell lung cancer patients and 30 breast cancer patients. miRNA levels were normalized to spiked-in cel-miR-54-5p and represented in scatter plots. Data are shown as means ±SE, *** P<0.001.

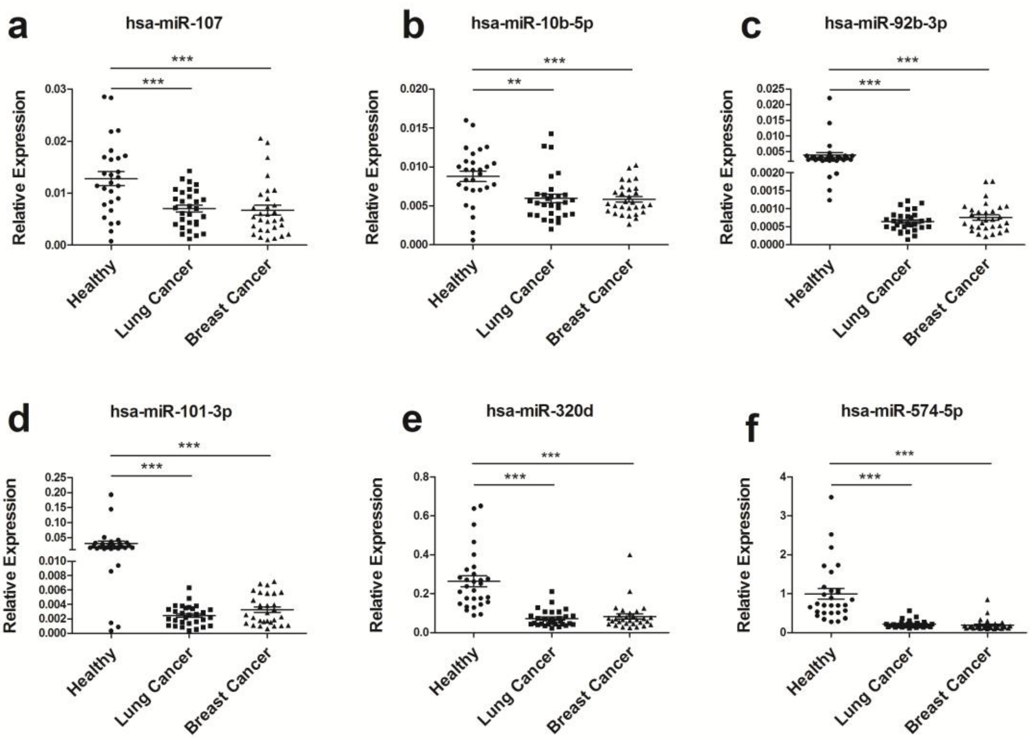

Supplement: Supplementary Figure 3 [file srep35611-s3.pdf]
