# Supplementary material for: Identification of reference genes for circulating microRNA analysis in colorectal cancer
Source: Sci Rep. 2016 Oct 19;6:35611. doi: 10.1038/srep35611 (PMC5069661; doi:10.1038/srep35611)
Supplement: Supplementary Table 1 [file srep35611-s4.pdf]

Identification of reference genes for circulating microRNA analysis in colorectal cancer

Yanqin Niu[1, 2*], Yike Wu[1*], Jinyong Huang[1], Qing Li[1], Kang Kang[3], Junle Qu[2], Furong Li[4#] and Deming Gou[1#]

Supplemental table 1 Primers and probe sequences in this study.

miRNA	Sequence (5'-3')	Forward primer	RT primer	Group in RT
>hsa-miR-92b-3p MIMAT0003218	UAUUGCACUCGUCCCGGCCUCC	TGGTATTGCACTCGTCCCG	GTGCAGGGTCCGAGGTCAGAGCCACCTGGGCAATTTTTTTTTTTTGGAGGC	1
>hsa-miR-490-3p MIMAT0002806	CAACCUGGAGGACUCCAUGCUG	GGCAACCTGGAGGACTCC	GTGCAGGGTCCGAGGTCAGAGCCACCTGGGCAATTTTTTTTTTTTCAGCAT	1
>hsa-miR-107 MIMAT0000104	AGCAGCAUUGUACAGGGCUAUCA	GGAGCAGCATTGTACAGGG	GTGCAGGGTCCGAGGTCAGAGCCACCTGGGCAATTTTTTTTTTTTGATAGC	1
>hsa-miR-30e-5p MIMAT0000692	UGUAAACAUCCUUGACUGGAAG	TTCGGTGTAAACATCCTTGAC	GTGCAGGGTCCGAGGTCAGAGCCACCTGGGCAATTTTTTTTTTTTCTTCCA	1
>hsa-miR-485-5p MIMAT0002175	AGAGGCUGGCCGUGAUGAAUUC	GGAGAGGCTGGCCGTGAT	GTGCAGGGTCCGAGGTCAGAGCCACCTGGGCAATTTTTTTTTTTTGAATTC	1
>hsa-miR-1237-3p MIMAT0005592	UCCUUCUGCUCCGUCCCCCAG	CGGTCCTTCTGCTCCGTC	GTGCAGGGTCCGAGGTCAGAGCCACCTGGGCAATTTTTTTTTTTTCTGGGG	1
>hsa-miR-372-3p MIMAT0000724	AAAGUGCUGCGACAUUUGAGCGU	CGGAAAGTGCTGCGACATTT	GTGCAGGGTCCGAGGTCAGAGCCACCTGGGCAATTTTTTTTTTTTACGCTC	1
>hsa-miR-744-5p MIMAT0004945	UGCGGGGCUAGGGCUAACAGCA	TGTGCGGGGCTAGGGCTA	GTGCAGGGTCCGAGGTCAGAGCCACCTGGGCAATTTTTTTTTTTTGCTGTT	2
>hsa-miR-3662 MIMAT0018083	GAAAAUGAUGAGUAGUGACUGAUG	CGGGAAAATGATGAGTAGTGA	GTGCAGGGTCCGAGGTCAGAGCCACCTGGGCAATTTTTTTTTTTTCATCAG	2
>hsa-miR-206 MIMAT0000462	UGGAAUGUAAGGAAGUGUGUGG	TTCGGTGGAATGTAAGGAAGT	GTGCAGGGTCCGAGGTCAGAGCCACCTGGGCAATTTTTTTTTTTTCCACAC	2
>hsa-miR-125a-3p MIMAT0004602	ACAGGUGAGGUUCUUGGGAGCC	CGGACAGGTGAGGTTCTTG	GTGCAGGGTCCGAGGTCAGAGCCACCTGGGCAATTTTTTTTTTTTGGCTCC	2
>hsa-miR-637 MIMAT0003307	ACUGGGGGCUUUCGGGCUCUGCGU	ACTGGGGGCTTTCGGGCT	GTGCAGGGTCCGAGGTCAGAGCCACCTGGGCAATTTTTTTTTTTTACGCAG	2
>hsa-miR-34c-3p MIMAT0004677	AAUCACUAACCACACGGCCAGG	TCGGAATCACTAACCACACG	GTGCAGGGTCCGAGGTCAGAGCCACCTGGGCAATTTTTTTTTTTTCCTGGC	2
>hsa-miR-877-3p MIMAT0004950	UCCUCUUCUCCCUCCUCCCAG	CGGTCCTCTTCTCCCTCC	GTGCAGGGTCCGAGGTCAGAGCCACCTGGGCAATTTTTTTTTTTTCTGGGA	2
>hsa-miR-200b-3p MIMAT0000318	UAAUACUGCCUGGUAAUGAUGA	TTCGGTAATACTGCCTGGTAA	GTGCAGGGTCCGAGGTCAGAGCCACCTGGGCAATTTTTTTTTTTTCATCAT	3
>hsa-miR-193b-3p MIMAT0002819	AACUGGCCCUCAAAGUCCCGCU	CGGAACTGGCCCTCAAAGT	GTGCAGGGTCCGAGGTCAGAGCCACCTGGGCAATTTTTTTTTTTTAGCGGG	3
>hsa-miR-378a-3p MIMAT0000732	ACUGGACUUGGAGUCAGAAGGC	CGGACTGGACTTGGAGTCA	GTGCAGGGTCCGAGGTCAGAGCCACCTGGGCAATTTTTTTTTTTTGCCTTC	3
>hsa-miR-4323 MIMAT0016875	CAGCCCCACAGCCUCAGA	GTCGGCAGCCCCACAGC	GTGCAGGGTCCGAGGTCAGAGCCACCTGGGCAATTTTTTTTTTTTCTGAGG	3
>hsa-miR-502-3p MIMAT0004775	AAUGCACCUGGGCAAGGAUUCA	TGGAATGCACCTGGGCAAG	GTGCAGGGTCCGAGGTCAGAGCCACCTGGGCAATTTTTTTTTTTTGAATCC	3
>hsa-miR-335-5p MIMAT0000765	UCAAGAGCAAUAACGAAAAAUGU	TCGGTCAAGAGCAATAACGAA	GTGCAGGGTCCGAGGTCAGAGCCACCTGGGCAATTTTTTTTTTTTACATTT	3
>hsa-miR-296-5p MIMAT0000690	AGGGCCCCCCCUCAAUCCUGU	GGAGGGCCCCCCCTCAA	GTGCAGGGTCCGAGGTCAGAGCCACCTGGGCAATTTTTTTTTTTTACAGGA	3
>hsa-miR-7-1-3p MIMAT0004553	CAACAAAUCACAGUCUGCCAUA	TTCGGCAACAAATCACAGTCT	GTGCAGGGTCCGAGGTCAGAGCCACCTGGGCAATTTTTTTTTTTTATGGCA	4
>hsa-miR-103b MIMAT0007402	UCAUAGCCCUGUACAAUGCUGCU	CGGTCATAGCCCTGTACAAT	GTGCAGGGTCCGAGGTCAGAGCCACCTGGGCAATTTTTTTTTTTTAGCAGC	4
>hsa-miR-1231 MIMAT0005586	GUGUCUGGGCGGACAGCUGC	CGGGTGTCTGGGCGGAC	GTGCAGGGTCCGAGGTCAGAGCCACCTGGGCAATTTTTTTTTTTTGCAGCT	4
>hsa-miR-625-3p MIMAT0004808	GACUAUAGAACUUUCCCCCUCA	TTCGGGACTATAGAACTTTCC	GTGCAGGGTCCGAGGTCAGAGCCACCTGGGCAATTTTTTTTTTTTGAGGGG	4
>hsa-miR-155-5p MIMAT0000646	UUAAUGCUAAUCGUGAUAGGGGU	TTCGGTTAATGCTAATCGTGAT	GTGCAGGGTCCGAGGTCAGAGCCACCTGGGCAATTTTTTTTTTTTACCCCT	4
>hsa-miR-1225-3p MIMAT0005573	UGAGCCCCUGUGCCGCCCCCAG	CGGTGAGCCCCTGTGCC	GTGCAGGGTCCGAGGTCAGAGCCACCTGGGCAATTTTTTTTTTTTCTGGGG	4
>hsa-miR-184 MIMAT0000454	UGGACGGAGAACUGAUAAGGGU	CGGTGGACGGAGAACTGAT	GTGCAGGGTCCGAGGTCAGAGCCACCTGGGCAATTTTTTTTTTTTACCCTT	4
>hsa-miR-34c-5p MIMAT0000686	AGGCAGUGUAGUUAGCUGAUUGC	TGGAGGCAGTGTAGTTAGCT	GTGCAGGGTCCGAGGTCAGAGCCACCTGGGCAATTTTTTTTTTTTGCAATC	5
>hsa-miR-3141 MIMAT0015010	GAGGGCGGGUGGAGGAGGA	TCGGGAGGGCGGGTGGA	GTGCAGGGTCCGAGGTCAGAGCCACCTGGGCAATTTTTTTTTTTTCCTCCT	5
>hsa-miR-330-3p MIMAT0000751	GCAAAGCACACGGCCUGCAGAGA	GGCAAAGCACACGGCCTG	GTGCAGGGTCCGAGGTCAGAGCCACCTGGGCAATTTTTTTTTTTTCTCTGC	5
>hsa-miR-223-5p MIMAT0004570	CGUGUAUUUGACAAGCUGAGUU	TCGGCGTGTATTTGACAAGC	GTGCAGGGTCCGAGGTCAGAGCCACCTGGGCAATTTTTTTTTTTTAACTCA	5
>hsa-miR-422a MIMAT0001339	ACUGGACUUAGGGUCAGAAGGC	CGGACTGGACTTAGGGTCA	GTGCAGGGTCCGAGGTCAGAGCCACCTGGGCAATTTTTTTTTTTTGCCTTC	5
>hsa-miR-151a-5p MIMAT0004697	UCGAGGAGCUCACAGUCUAGU	CGGTCGAGGAGCTCACAG	GTGCAGGGTCCGAGGTCAGAGCCACCTGGGCAATTTTTTTTTTTTACTAGA	5
>hsa-miR-182-5p MIMAT0000259	UUUGGCAAUGGUAGAACUCACACU	TGGTTTGGCAATGGTAGAACT	GTGCAGGGTCCGAGGTCAGAGCCACCTGGGCAATTTTTTTTTTTTAGTGTG	5
>hsa-miR-339-5p MIMAT0000764	UCCCUGUCCUCCAGGAGCUCACG	GTCCCTGTCCTCCAGGAG	GTGCAGGGTCCGAGGTCAGAGCCACCTGGGCAATTTTTTTTTTTTCGTGAG	6
>hsa-miR-100-5p MIMAT0000098	AACCCGUAGAUCCGAACUUGUG	CGGAACCCGTAGATCCGAA	GTGCAGGGTCCGAGGTCAGAGCCACCTGGGCAATTTTTTTTTTTTCACAAG	6

>hsa-miR-23b-5p MIMAT0004587	UGGGUUCCUGGCAUGCUGAUUU	GGTGGGTTCCTGGCATGC	GTGCAGGGTCCGAGGTCAGAGCCACCTGGGCAATTTTTTTTTTTAAATCA	6
>hsa-miR-607 MIMAT0003275	GUUCAAAUCCAGAUCUAUAAC	GTCGGGTTCAAATCCAGATC	GTGCAGGGTCCGAGGTCAGAGCCACCTGGGCAATTTTTTTTTTTGTTATA	6
>hsa-miR-219a-5p MIMAT0000276	UGAUUGUCCAAACGCAAUUCU	TTCGGTGATTGTCCAAACGC	GTGCAGGGTCCGAGGTCAGAGCCACCTGGGCAATTTTTTTTTTTAGAATT	6
>hsa-miR-374a-5p MIMAT0000727	UUAUAAUACAACCUGAUAAGUG	TGTCGGTTATAATACAACCTGA	GTGCAGGGTCCGAGGTCAGAGCCACCTGGGCAATTTTTTTTTTTCACTTA	6
>hsa-miR-4651 MIMAT0019715	CGGGGUGGGUGAGGUCGGGC	TGGCGGGGTGGGTGAGG	GTGCAGGGTCCGAGGTCAGAGCCACCTGGGCAATTTTTTTTTTTGCCCGA	6
>hsa-miR-335-3p MIMAT0004703	UUUUUCAUUAUUGCUCCUGACC	TGTCGGTTTTTCATTATTGCTC	GTGCAGGGTCCGAGGTCAGAGCCACCTGGGCAATTTTTTTTTTTGGTCAG	7
>hsa-miR-196b-3p MIMAT0009201	UCGACAGCACGACACUGCCUUC	TGGTCGACAGCACGACACT	GTGCAGGGTCCGAGGTCAGAGCCACCTGGGCAATTTTTTTTTTTGAAGGC	7
>hsa-miR-92a-2-5p MIMAT0004508	GGGUGGGGAUUUGUUGCAUUAC	TGGGGGTGGGGATTTGTTG	GTGCAGGGTCCGAGGTCAGAGCCACCTGGGCAATTTTTTTTTTTGTAATG	7
>hsa-miR-4258 MIMAT0016879	CCCCGCCACCGCCUUGG	GTCGGCCCCGCCACCG	GTGCAGGGTCCGAGGTCAGAGCCACCTGGGCAATTTTTTTTTTTCCAAGG	7
>hsa-miR-3653-3p MIMAT0018073	CUAAGAAGUUGACUGAAG	TCGGTCGGCTAAGAAGTTGA	GTGCAGGGTCCGAGGTCAGAGCCACCTGGGCAATTTTTTTTTTTCTTCAG	7
>hsa-miR-4286 MIMAT0016916	ACCCCACUCCUGGUACC	TGGTCGGACCCCACTCCT	GTGCAGGGTCCGAGGTCAGAGCCACCTGGGCAATTTTTTTTTTTGGTACC	7
>hsa-miR-9-5p MIMAT0000441	UCUUUGGUUAUCUAGCUGUAUGA	TCGGTCTTTGGTTATCTAGCT	GTGCAGGGTCCGAGGTCAGAGCCACCTGGGCAATTTTTTTTTTTCATACA	7
>hsa-miR-329-3p MIMAT0001629	AACACACCUGGUUAACCUCUUU	TCGGAACACACCTGGTTAAC	GTGCAGGGTCCGAGGTCAGAGCCACCTGGGCAATTTTTTTTTTTAAAGAG	8
>hsa-miR-361-5p MIMAT0000703	UUAUCAGAAUCUCCAGGGGUAC	TTCGGTTATCAGAATCTCCAG	GTGCAGGGTCCGAGGTCAGAGCCACCTGGGCAATTTTTTTTTTTGTACCC	8
>hsa-miR-199a-5p MIMAT0000231	CCCAGUGUUCAGACUACCUGUUC	GGCCCAGTGTTCAGACTAC	GTGCAGGGTCCGAGGTCAGAGCCACCTGGGCAATTTTTTTTTTTGAACAG	8
>hsa-miR-3120-3p MIMAT0014982	CACAGCAAGUGUAGACAGGCA	TTCGGCACAGCAAGTGTAGA	GTGCAGGGTCCGAGGTCAGAGCCACCTGGGCAATTTTTTTTTTTGCCTGT	8
>hsa-miR-126-5p MIMAT0000444	CAUUAUUACUUUUGGUACGCG	GGTCGGCATTATTACTTTTGG	GTGCAGGGTCCGAGGTCAGAGCCACCTGGGCAATTTTTTTTTTTCGCGTA	8
>hsa-miR-4301 MIMAT0016850	UCCCACUACUUCACUUGUGA	GTCGGTCCCACTACTTCAC	GTGCAGGGTCCGAGGTCAGAGCCACCTGGGCAATTTTTTTTTTTCACAAG	8
>hsa-miR-503-5p MIMAT0002874	UAGCAGCGGGAACAGUUCUGCAG	GGTAGCAGCGGGAACAGTT	GTGCAGGGTCCGAGGTCAGAGCCACCTGGGCAATTTTTTTTTTTCTGCAG	8
>hsa-miR-485-3p MIMAT0002176	GUCAUACACGGCUCUCCUCUCU	TGGGTCATACACGGCTCTC	GTGCAGGGTCCGAGGTCAGAGCCACCTGGGCAATTTTTTTTTTTAGAGAG	9
>hsa-miR-18a-5p MIMAT0000072	UAAGGUGCAUCUAGUGCAGAUAG	TGGTAAGGTGCATCTAGTGC	GTGCAGGGTCCGAGGTCAGAGCCACCTGGGCAATTTTTTTTTTTCTATCT	9
>hsa-miR-1277-3p MIMAT0005933	UACGUAGAUAUAUAUGUAUUUU	TGTCGGTACGTAGATATATATG	GTGCAGGGTCCGAGGTCAGAGCCACCTGGGCAATTTTTTTTTTTAAAATA	9
>hsa-miR-654-5p MIMAT0003330	UGGUGGGCCGCAGAACAUGUGC	TGTGGTGGGCCGCAGAAC	GTGCAGGGTCCGAGGTCAGAGCCACCTGGGCAATTTTTTTTTTTGCACAT	9
>hsa-let-7b-3p MIMAT0004482	CUAUACAACCUACUGCCUUCCC	TCGGCTATACAACCTACTGC	GTGCAGGGTCCGAGGTCAGAGCCACCTGGGCAATTTTTTTTTTTGGGAAG	9
>hsa-miR-375 MIMAT0000728	UUUGUUCGUUCGGCUCGCGUGA	CGGTTTGTTCGTTCGGCTC	GTGCAGGGTCCGAGGTCAGAGCCACCTGGGCAATTTTTTTTTTTCACGCG	9
>hsa-miR-365a-3p MIMAT0000710	UAAUGCCCCUAAAAAUCCUUAU	GTCGGTAATGCCCCTAAAAAT	GTGCAGGGTCCGAGGTCAGAGCCACCTGGGCAATTTTTTTTTTTATAAGG	9
>hsa-miR-3135b MIMAT0018985	GGCUGGAGCGAGUGCAGUGGUG	TGGGCTGGAGCGAGTGCA	GTGCAGGGTCCGAGGTCAGAGCCACCTGGGCAATTTTTTTTTTTCACCAC	10
>hsa-miR-373-5p MIMAT0000725	ACUCAAAAUGGGGGCGCUUUCC	TGGACTCAAAATGGGGGCG	GTGCAGGGTCCGAGGTCAGAGCCACCTGGGCAATTTTTTTTTTTGGAAAG	10
>hsa-miR-424-3p MIMAT0004749	CAAAACGUGAGGCGCUGCUAU	CGGCAAAACGTGAGGCGC	GTGCAGGGTCCGAGGTCAGAGCCACCTGGGCAATTTTTTTTTTTATAGCA	10
>hsa-miR-181c-5p MIMAT0000258	AACAUUCAACCUGUCGGUGAGU	TCGGAACATTCAACCTGTCG	GTGCAGGGTCCGAGGTCAGAGCCACCTGGGCAATTTTTTTTTTTACTCAC	10
>hsa-miR-4454 MIMAT0018976	GGAUCCGAGUCACGGCACCA	TCGGGGATCCGAGTCACG	GTGCAGGGTCCGAGGTCAGAGCCACCTGGGCAATTTTTTTTTTTGGTGCC	10
>hsa-miR-379-5p MIMAT0000733	UGGUAGACUAUGGAACGUAGG	GTCGGTGGTAGACTATGGAA	GTGCAGGGTCCGAGGTCAGAGCCACCTGGGCAATTTTTTTTTTTCCTACG	10
>hsa-miR-361-3p MIMAT0004682	UCCCCCAGGUGUGAUUCUGAUUU	TGTCCCCCAGGTGTGATTC	GTGCAGGGTCCGAGGTCAGAGCCACCTGGGCAATTTTTTTTTTTAAATCA	10
>hsa-miR-499a-5p MIMAT0002870	UUAAGACUUGCAGUGAUGUUU	GTCGGTTAAGACTTGCAGTG	GTGCAGGGTCCGAGGTCAGAGCCACCTGGGCAATTTTTTTTTTTAAACAT	11
>hsa-miR-302b-5p MIMAT0000714	ACUUUAACAUGGAAGUGCUUUC	GTCGGACTTTAACATGGAAGT	GTGCAGGGTCCGAGGTCAGAGCCACCTGGGCAATTTTTTTTTTTGAAAGC	11
>hsa-miR-190a-5p MIMAT0000458	UGAUAUGUUUGAUAUAUUAGGU	GGTCGGTGATATGTTTGATATA	GTGCAGGGTCCGAGGTCAGAGCCACCTGGGCAATTTTTTTTTTTACCTAA	11
>hsa-miR-19b-1-5p MIMAT0004491	AGUUUUGCAGGUUUGCAUCCAGC	CGGAGTTTTGCAGGTTTGCA	GTGCAGGGTCCGAGGTCAGAGCCACCTGGGCAATTTTTTTTTTTGCTGGA	11
>hsa-miR-1246 MIMAT0005898	AAUGGAUUUUUGGAGCAGG	CGGTCGGAATGGATTTTTGG	GTGCAGGGTCCGAGGTCAGAGCCACCTGGGCAATTTTTTTTTTTCCTGCT	11
>hsa-miR-382-5p MIMAT0000737	GAAGUUGUUCGUGGUGGAUUCG	CGGGAAGTTGTTCGTGGTG	GTGCAGGGTCCGAGGTCAGAGCCACCTGGGCAATTTTTTTTTTTCGAATC	11
>hsa-miR-598-3p MIMAT0003266	UACGUCAUCGUUGUCAUCGUCA	TCGGTACGTCATCGTTGTCA	GTGCAGGGTCCGAGGTCAGAGCCACCTGGGCAATTTTTTTTTTTGACGAT	11
>hsa-miR-16-5p MIMAT0000069	UAGCAGCACGUAAAUAUUGGCG	TTCGGTAGCAGCACGTAAATA	GTGCAGGGTCCGAGGTCAGAGCCACCTGGGCAATTTTTTTTTTTCGCCAA	12
>hsa-miR-421 MIMAT0003339	AUCAACAGACAUUAAUUGGGCGC	GTCGGATCAACAGACATTAATT	GTGCAGGGTCCGAGGTCAGAGCCACCTGGGCAATTTTTTTTTTTGCGCCC	12
>hsa-miR-29a-5p MIMAT0004503	ACUGAUUUCUUUUGGUGUUCAG	GTCGGACTGATTTCTTTTGGT	GTGCAGGGTCCGAGGTCAGAGCCACCTGGGCAATTTTTTTTTTTCTGAAC	12

>hsa-miR-195-5p MIMAT0000461	UAGCAGCACAGAAAUAUUGGC	GTCGGTAGCAGCACAGAAAT	GTGCAGGGTCCGAGGTCAGAGCCACCTGGGCAATTTTTTTTTTTGCCAAT	12
>hsa-miR-125a-5p MIMAT0000443	UCCCUGAGACCCUUUAACCUGUGA	TGTCCCTGAGACCCTTTAAC	GTGCAGGGTCCGAGGTCAGAGCCACCTGGGCAATTTTTTTTTTTCACAGG	12
>hsa-miR-323a-5p MIMAT0004696	AGGUGGUCCGUGGCGCGUUCGC	GAGGTGGTCCGTGGCGC	GTGCAGGGTCCGAGGTCAGAGCCACCTGGGCAATTTTTTTTTTTGCGAAC	12
>hsa-miR-7-2-3p MIMAT0004554	CAACAAAUCCCAGUCUACCUAA	TCGGCAACAAATCCCAGTCT	GTGCAGGGTCCGAGGTCAGAGCCACCTGGGCAATTTTTTTTTTTAGGTAG	12
>hsa-miR-193b-5p MIMAT0004767	CGGGGUUUUGAGGGCGAGAUGA	TGCGGGGTTTTGAGGGCG	GTGCAGGGTCCGAGGTCAGAGCCACCTGGGCAATTTTTTTTTTTCATCTC	13
>hsa-miR-371b-5p MIMAT0019892	ACUCAAAAGAUGGCGGCACUUU	CGGACTCAAAAGATGGCGG	GTGCAGGGTCCGAGGTCAGAGCCACCTGGGCAATTTTTTTTTTTAAAGTG	13
>hsa-miR-219a-1-3p MIMAT0004567	AGAGUUGAGUCUGGACGUCCCG	CGGAGAGTTGAGTCTGGAC	GTGCAGGGTCCGAGGTCAGAGCCACCTGGGCAATTTTTTTTTTTCGGGAC	13
>hsa-miR-378b MIMAT0014999	ACUGGACUUGGAGGCAGAA	TGTCGGACTGGACTTGGAG	GTGCAGGGTCCGAGGTCAGAGCCACCTGGGCAATTTTTTTTTTTCTGCCT	13
>hsa-miR-214-3p MIMAT0000271	ACAGCAGGCACAGACAGGCAGU	TGGACAGCAGGCACAGACA	GTGCAGGGTCCGAGGTCAGAGCCACCTGGGCAATTTTTTTTTTTACTGCC	13
>hsa-miR-217 MIMAT0000274	UACUGCAUCAGGAACUGAUUGGA	TGGTACTGCATCAGGAACTG	GTGCAGGGTCCGAGGTCAGAGCCACCTGGGCAATTTTTTTTTTTCCAATC	13
>hsa-miR-3689a-5p MIMAT0018117	UGUGAUAUCAUGGUUCCUGGGA	TTCGGTGTGATATCATGGTTC	GTGCAGGGTCCGAGGTCAGAGCCACCTGGGCAATTTTTTTTTTTCCCAGG	13
>hsa-miR-3163 MIMAT0015037	UAUAAAAUGAGGGCAGUAAGAC	TTCGGTATAAAATGAGGGCAG	GTGCAGGGTCCGAGGTCAGAGCCACCTGGGCAATTTTTTTTTTTGTCTTA	14
>hsa-let-7f-2-3p MIMAT0004487	CUAUACAGUCUACUGUCUUUCC	TTCGGCTATACAGTCTACTGT	GTGCAGGGTCCGAGGTCAGAGCCACCTGGGCAATTTTTTTTTTTGGAAAG	14
>hsa-miR-3622a-5p MIMAT0018003	CAGGCACGGGAGCUCAGGUGAG	TGCAGGCACGGGAGCTCA	GTGCAGGGTCCGAGGTCAGAGCCACCTGGGCAATTTTTTTTTTTCTCACC	14
>hsa-miR-1290 MIMAT0005880	UGGAUUUUUGGAUCAGGGA	CGGTCGGTGGATTTTTGGAT	GTGCAGGGTCCGAGGTCAGAGCCACCTGGGCAATTTTTTTTTTTCCCTGA	14
>hsa-miR-1976 MIMAT0009451	CCUCCUGCCCUCCUUGCUGU	CGGCCTCCTGCCCTCCT	GTGCAGGGTCCGAGGTCAGAGCCACCTGGGCAATTTTTTTTTTTACAGCA	14
>hsa-miR-339-3p MIMAT0004702	UGAGCGCCUCGACGACAGAGCCG	GTGAGCGCCTCGACGACA	GTGCAGGGTCCGAGGTCAGAGCCACCTGGGCAATTTTTTTTTTTCGGCTC	14
>hsa-miR-1287-5p MIMAT0005878	UGCUGGAUCAGUGGUUCGAGUC	CGGTGCTGGATCAGTGGTT	GTGCAGGGTCCGAGGTCAGAGCCACCTGGGCAATTTTTTTTTTTGACTCG	14
>hsa-miR-2467-3p MIMAT0019953	AGCAGAGGCAGAGAGGCUCAGG	GGAGCAGAGGCAGAGAGG	GTGCAGGGTCCGAGGTCAGAGCCACCTGGGCAATTTTTTTTTTTCCTGAG	15
>hsa-miR-3176 MIMAT0015053	ACUGGCCUGGGACUACCGG	TTCGGACTGGCCTGGGAC	GTGCAGGGTCCGAGGTCAGAGCCACCTGGGCAATTTTTTTTTTTCCGGTA	15
>hsa-miR-519b-5p MIMAT0005454	CUCUAGAGGGAAGCGCUUUCUG	GGCTCTAGAGGGAAGCGC	GTGCAGGGTCCGAGGTCAGAGCCACCTGGGCAATTTTTTTTTTTCAGAAA	15
>hsa-miR-202-3p MIMAT0002811	AGAGGUAUAGGGCAUGGGAA	GTCGGAGAGGTATAGGGCA	GTGCAGGGTCCGAGGTCAGAGCCACCTGGGCAATTTTTTTTTTTCCCATG	15
>hsa-miR-3074-3p MIMAT0015027	GAUAUCAGCUCAGUAGGCACCG	TCGGGATATCAGCTCAGTAG	GTGCAGGGTCCGAGGTCAGAGCCACCTGGGCAATTTTTTTTTTTCGGTGC	15
>hsa-miR-1260a MIMAT0005911	AUCCCACCUCUGCCACCA	GGTCGGATCCCACCTCTG	GTGCAGGGTCCGAGGTCAGAGCCACCTGGGCAATTTTTTTTTTTGGTGGC	15
>hsa-miR-19b-2-5p MIMAT0004492	AGUUUUGCAGGUUUGCAUUUCA	TCGGAGTTTTGCAGGTTTGC	GTGCAGGGTCCGAGGTCAGAGCCACCTGGGCAATTTTTTTTTTTGAAATG	15
>hsa-miR-143-3p MIMAT0000435	UGAGAUGAAGCACUGUAGCUC	TTCGGTGAGATGAAGCACTG	GTGCAGGGTCCGAGGTCAGAGCCACCTGGGCAATTTTTTTTTTTGAGCTA	16
>hsa-miR-410-3p MIMAT0002171	AAUAUAACACAGAUGGCCUGU	GGTCGGAATATAACACAGATG	GTGCAGGGTCCGAGGTCAGAGCCACCTGGGCAATTTTTTTTTTTACAGGC	16
>hsa-miR-551b-3p MIMAT0003233	GCGACCCAUACUUGGUUUCAG	CGGGCGACCCATACTTGG	GTGCAGGGTCCGAGGTCAGAGCCACCTGGGCAATTTTTTTTTTTCTGAAA	16
>hsa-miR-27a-3p MIMAT0000084	UUCACAGUGGCUAAGUUCCGC	TTCGGTTCACAGTGGCTAAG	GTGCAGGGTCCGAGGTCAGAGCCACCTGGGCAATTTTTTTTTTTGCGGAA	16
>hsa-miR-1180-3p MIMAT0005825	UUUCCGGCUCGCGUGGGUGUGU	TGTTTCCGGCTCGCGTGG	GTGCAGGGTCCGAGGTCAGAGCCACCTGGGCAATTTTTTTTTTTACACAC	16
>hsa-miR-4423-5p MIMAT0019232	AGUUGCCUUUUUGUUCCCAUGC	TTCGGAGTTGCCTTTTTGTTC	GTGCAGGGTCCGAGGTCAGAGCCACCTGGGCAATTTTTTTTTTTGCATGG	16
>hsa-miR-423-5p MIMAT0004748	UGAGGGGCAGAGAGCGAGACUUU	GTGAGGGGCAGAGAGCGA	GTGCAGGGTCCGAGGTCAGAGCCACCTGGGCAATTTTTTTTTTTAAAGTC	16
>hsa-miR-615-5p MIMAT0004804	GGGGGUCCCCGGUGCUCGGAUC	TGGGGGTCCCCGGTGCT	GTGCAGGGTCCGAGGTCAGAGCCACCTGGGCAATTTTTTTTTTTGATCCG	17
>hsa-miR-185-5p MIMAT0000455	UGGAGAGAAAGGCAGUUCCUGA	CGGTGGAGAGAAAGGCAGT	GTGCAGGGTCCGAGGTCAGAGCCACCTGGGCAATTTTTTTTTTTCAGGAA	17
>hsa-miR-181b-5p MIMAT0000257	AACAUUCAUUGCUGUCGGUGGGU	CGGAACATTCATTGCTGTCG	GTGCAGGGTCCGAGGTCAGAGCCACCTGGGCAATTTTTTTTTTTACCCAC	17
>hsa-miR-7703 MIMAT0030018	UUGCACUCUGGCCUUCUCCCAGG	GGTTGCACTCTGGCCTTCT	GTGCAGGGTCCGAGGTCAGAGCCACCTGGGCAATTTTTTTTTTTCCTGGG	17
>hsa-miR-148b-3p MIMAT0000759	UCAGUGCAUCACAGAACUUUGU	TCGGTCAGTGCATCACAGAA	GTGCAGGGTCCGAGGTCAGAGCCACCTGGGCAATTTTTTTTTTTACAAAG	17
>hsa-miR-25-3p MIMAT0000081	CAUUGCACUUGUCUCGGUCUGA	CGGCATTGCACTTGTCTCG	GTGCAGGGTCCGAGGTCAGAGCCACCTGGGCAATTTTTTTTTTTCAGACC	17
>hsa-miR-99b-5p MIMAT0000689	CACCCGUAGAACCGACCUUGCG	GGCACCCGTAGAACCGAC	GTGCAGGGTCCGAGGTCAGAGCCACCTGGGCAATTTTTTTTTTTCGCAAG	17
>hsa-miR-628-3p MIMAT0003297	UCUAGUAAGAGUGGCAGUCGA	TTCGGTCTAGTAAGAGTGGC	GTGCAGGGTCCGAGGTCAGAGCCACCTGGGCAATTTTTTTTTTTCGACTG	18
>hsa-miR-151b MIMAT0010214	UCGAGGAGCUCACAGUCU	GGTCGGTCGAGGAGCTCA	GTGCAGGGTCCGAGGTCAGAGCCACCTGGGCAATTTTTTTTTTTAGACTG	18
>hsa-miR-6876-5p MIMAT0027652	CAGGAAGGAGACAGGCAGUUCA	GGCAGGAAGGAGACAGGC	GTGCAGGGTCCGAGGTCAGAGCCACCTGGGCAATTTTTTTTTTTGAACTG	18
>hsa-miR-550a-5p MIMAT0004800	AGUGCCUGAGGGAGUAAGAGCCC	GGAGTGCCTGAGGGAGTAA	GTGCAGGGTCCGAGGTCAGAGCCACCTGGGCAATTTTTTTTTTTGGGCTC	18

>hsa-miR-505-3p MIMAT0002876	CGUCAACACUUGCUGGUUUCCU	TGGCGTCAACACTTGCTGG	GTGCAGGGTCCGAGGTCAGAGCCACCTGGGCAATTTTTTTTTTTAGGAAA	18
>hsa-miR-205-5p MIMAT0000266	UCCUUCAUUCCACCGGAGUCUG	TGGTCCTTCATTCCACCGG	GTGCAGGGTCCGAGGTCAGAGCCACCTGGGCAATTTTTTTTTTTCAGACT	18
>hsa-miR-224-5p MIMAT0000281	CAAGUCACUAGUGGUUCCGUU	TTCGGCAAGTCACTAGTGGT	GTGCAGGGTCCGAGGTCAGAGCCACCTGGGCAATTTTTTTTTTTAACGGA	18
>hsa-miR-138-5p MIMAT0000430	AGCUGGUGUUGUGAAUCAGGCCG	TGGAGCTGGTGTTGTGAATC	GTGCAGGGTCCGAGGTCAGAGCCACCTGGGCAATTTTTTTTTTTCGGCCT	19
>hsa-miR-340-3p MIMAT0000750	UCCGUCUCAGUUACUUUAUAGC	TCGGTCCGTCTCAGTTACTT	GTGCAGGGTCCGAGGTCAGAGCCACCTGGGCAATTTTTTTTTTTGCTATA	19
>hsa-miR-874-3p MIMAT0004911	CUGCCCUGGCCCGAGGGACCGA	TCTGCCCTGGCCCGAGG	GTGCAGGGTCCGAGGTCAGAGCCACCTGGGCAATTTTTTTTTTTCGGTCC	19
>hsa-miR-1537-3p MIMAT0007399	AAAACCGUCUAGUUACAGUUGU	TTCGGAAAACCGTCTAGTTAC	GTGCAGGGTCCGAGGTCAGAGCCACCTGGGCAATTTTTTTTTTTACAACT	19
>hsa-miR-145-3p MIMAT0004601	GGAUUCCUGGAAAUACUGUUCU	TCGGGGATTCCTGGAAATAC	GTGCAGGGTCCGAGGTCAGAGCCACCTGGGCAATTTTTTTTTTTAGAACA	19
>hsa-miR-652-3p MIMAT0003322	AAUGGCGCCACUAGGGUUGUG	CGGAATGGCGCCACTAGG	GTGCAGGGTCCGAGGTCAGAGCCACCTGGGCAATTTTTTTTTTTCACAAC	19
>hsa-miR-130a-3p MIMAT0000425	CAGUGCAAUGUUAAAAGGGCAU	GTCGGCAGTGCAATGTTAAAA	GTGCAGGGTCCGAGGTCAGAGCCACCTGGGCAATTTTTTTTTTTATGCCC	19
>hsa-miR-19a-3p MIMAT0000073	UGUGCAAAUCUAUGCAAAACUGA	TCGGTGTGCAAATCTATGCAA	GTGCAGGGTCCGAGGTCAGAGCCACCTGGGCAATTTTTTTTTTTCAGTTT	20
>hsa-miR-451a MIMAT0001631	AAACCGUUACCAUUACUGAGUU	TTCGGAAACCGTTACCATTAC	GTGCAGGGTCCGAGGTCAGAGCCACCTGGGCAATTTTTTTTTTTAACTCA	20
>hsa-miR-144-5p MIMAT0004600	GGAUAUCAUCAUAUACUGUAAG	GTCGGGGATATCATCATATAC	GTGCAGGGTCCGAGGTCAGAGCCACCTGGGCAATTTTTTTTTTTCTTACA	20
>hsa-miR-92a-3p MIMAT0000092	UAUUGCACUUGUCCCGGCCUGU	CGGTATTGCACTTGTCCCG	GTGCAGGGTCCGAGGTCAGAGCCACCTGGGCAATTTTTTTTTTTACAGGC	20
>hsa-miR-218-1-3p MIMAT0004565	AUGGUUCCGUCAAGCACCAUGG	CGGATGGTTCCGTCAAGCA	GTGCAGGGTCCGAGGTCAGAGCCACCTGGGCAATTTTTTTTTTTCCATGG	20
>hsa-miR-486-3p MIMAT0004762	CGGGGCAGCUCAGUACAGGAU	TGGCGGGGCAGCTCAGTA	GTGCAGGGTCCGAGGTCAGAGCCACCTGGGCAATTTTTTTTTTTATCCTG	20
>hsa-miR-627-5p MIMAT0003296	GUGAGUCUCUAAGAAAAGAGGA	TTCGGGTGAGTCTCTAAGAAA	GTGCAGGGTCCGAGGTCAGAGCCACCTGGGCAATTTTTTTTTTTCCTCTT	20
>hsa-miR-1183 MIMAT0005828	CACUGUAGGUGAUGGUGAGAGUGGGC[illegible]	CACTGTAGGTGATGGTGAGAG	GTGCAGGGTCCGAGGTCAGAGCCACCTGGGCAATTTTTTTTTTTGCCCAC	21
>hsa-miR-126-3p MIMAT0000445	UCGUACCGUGAGUAAUAAUGCG	TCGGTCGTACCGTGAGTAAT	GTGCAGGGTCCGAGGTCAGAGCCACCTGGGCAATTTTTTTTTTTCGCATT	21
>hsa-let-7c-5p MIMAT0000064	UGAGGUAGUAGGUUGUAUGGUU	TCGGTGAGGTAGTAGGTTGT	GTGCAGGGTCCGAGGTCAGAGCCACCTGGGCAATTTTTTTTTTTAACCAT	21
>hsa-miR-30b-5p MIMAT0000420	UGUAAACAUCCUACACUCAGCU	TTCGGTGTAAACATCCTACAC	GTGCAGGGTCCGAGGTCAGAGCCACCTGGGCAATTTTTTTTTTTAGCTGA	21
>hsa-miR-378i MIMAT0019074	ACUGGACUAGGAGUCAGAAGG	TCGGACTGGACTAGGAGTC	GTGCAGGGTCCGAGGTCAGAGCCACCTGGGCAATTTTTTTTTTTCCTTCT	21
>hsa-miR-3200-5p MIMAT0017392	AAUCUGAGAAGGCGCACAAGGU	CGGAATCTGAGAAGGCGCA	GTGCAGGGTCCGAGGTCAGAGCCACCTGGGCAATTTTTTTTTTTACCTTG	21
>hsa-miR-2355-3p MIMAT0017950	AUUGUCCUUGCUGUUUGGAGAU	TTCGGATTGTCCTTGCTGTTT	GTGCAGGGTCCGAGGTCAGAGCCACCTGGGCAATTTTTTTTTTTATCTCC	21
>hsa-miR-204-5p MIMAT0000265	UUCCCUUUGUCAUCCUAUGCCU	TCGGTTCCCTTTGTCATCCT	GTGCAGGGTCCGAGGTCAGAGCCACCTGGGCAATTTTTTTTTTTAGGCAT	22
>hsa-miR-26a-1-3p MIMAT0004499	CCUAUUCUUGGUUACUUGCACG	TTCGGCCTATTCTTGGTTACT	GTGCAGGGTCCGAGGTCAGAGCCACCTGGGCAATTTTTTTTTTTCGTGCA	22
>hsa-miR-18b-5p MIMAT0001412	UAAGGUGCAUCUAGUGCAGUUAG	TGGTAAGGTGCATCTAGTGC	GTGCAGGGTCCGAGGTCAGAGCCACCTGGGCAATTTTTTTTTTTCTAACT	22
>hsa-miR-20b-3p MIMAT0004752	ACUGUAGUAUGGGCACUUCCAG	CGGACTGTAGTATGGGCAC	GTGCAGGGTCCGAGGTCAGAGCCACCTGGGCAATTTTTTTTTTTCTGGAA	22
>hsa-miR-338-5p MIMAT0004701	AACAAUAUCCUGGUGCUGAGUG	TCGGAACAATATCCTGGTGC	GTGCAGGGTCCGAGGTCAGAGCCACCTGGGCAATTTTTTTTTTTCACTCA	22
>hsa-miR-517a-3p MIMAT0002852	AUCGUGCAUCCCUUUAGAGUGU	TCGGATCGTGCATCCCTTTA	GTGCAGGGTCCGAGGTCAGAGCCACCTGGGCAATTTTTTTTTTTACACTC	22
>hsa-miR-542-3p MIMAT0003389	UGUGACAGAUUGAUAACUGAAA	TGTCGGTGTGACAGATTGATA	GTGCAGGGTCCGAGGTCAGAGCCACCTGGGCAATTTTTTTTTTTCAGTTA	22
>hsa-miR-28-3p MIMAT0004502	CACUAGAUUGUGAGCUCCUGGA	TCGGCACTAGATTGTGAGCT	GTGCAGGGTCCGAGGTCAGAGCCACCTGGGCAATTTTTTTTTTTCCAGGA	23
>hsa-miR-4687-5p MIMAT0019774	CAGCCCUCCUCCCGCACCCAAA	TGCAGCCCTCCTCCCGC	GTGCAGGGTCCGAGGTCAGAGCCACCTGGGCAATTTTTTTTTTTGGGTGC	23
>hsa-miR-148a-3p MIMAT0000243	UCAGUGCACUACAGAACUUUGU	TCGGTCAGTGCACTACAGAA	GTGCAGGGTCCGAGGTCAGAGCCACCTGGGCAATTTTTTTTTTTACAAAG	23
>hsa-miR-10b-5p MIMAT0000254	UACCCUGUAGAACCGAAUUUGUG	TGGTACCCTGTAGAACCGAA	GTGCAGGGTCCGAGGTCAGAGCCACCTGGGCAATTTTTTTTTTTCACAAA	23
>hsa-miR-4296 MIMAT0016845	AUGUGGGCUCAGGCUCA	CGGTCGGATGTGGGCTCA	GTGCAGGGTCCGAGGTCAGAGCCACCTGGGCAATTTTTTTTTTTGAGCCT	23
>hsa-miR-10a-5p MIMAT0000253	UACCCUGUAGAUCCGAAUUUGUG	TGGTACCCTGTAGATCCGAA	GTGCAGGGTCCGAGGTCAGAGCCACCTGGGCAATTTTTTTTTTTCACAAA	23
>hsa-miR-19b-3p MIMAT0000074	UGUGCAAAUCCAUGCAAAACUGA	CGGTGTGCAAATCCATGCAA	GTGCAGGGTCCGAGGTCAGAGCCACCTGGGCAATTTTTTTTTTTCAGTTT	23
>hsa-miR-629-5p MIMAT0004810	UGGGUUUACGUUGGGAGAACU	TCGGTGGGTTTACGTTGGG	GTGCAGGGTCCGAGGTCAGAGCCACCTGGGCAATTTTTTTTTTTAGTTCT	24
>hsa-miR-4505 MIMAT0019041	AGGCUGGGCUGGGACGGA	GTCGGAGGCTGGGCTGG	GTGCAGGGTCCGAGGTCAGAGCCACCTGGGCAATTTTTTTTTTTCCGTCC	24
>hsa-miR-211-5p MIMAT0000268	UUCCCUUUGUCAUCCUUCGCCU	TCGGTTCCCTTTGTCATCCT	GTGCAGGGTCCGAGGTCAGAGCCACCTGGGCAATTTTTTTTTTTAGGCGA	24
>hsa-miR-1286 MIMAT0005877	UGCAGGACCAAGAUGAGCCCU	TCGGTGCAGGACCAAGATG	GTGCAGGGTCCGAGGTCAGAGCCACCTGGGCAATTTTTTTTTTTAGGGCT	24
>hsa-miR-532-5p MIMAT0002888	CAUGCCUUGAGUGUAGGACCGU	CGGCATGCCTTGAGTGTAG	GTGCAGGGTCCGAGGTCAGAGCCACCTGGGCAATTTTTTTTTTTACGGTC	24

>hsa-miR-450a-5p MIMAT0001545	UUUUGCGAUGUGUUCCUAAUAU	TCGGTTTTGCGATGTGTTCC	GTGCAGGGTCCGAGGTCAGAGCCACCTGGGCAATTTTTTTTTTTATATTA	24
>hsa-miR-139-3p MIMAT0004552	UGGAGACGCGGCCCUGUUGGAGU	TTGGAGACGCGGCCCTGT	GTGCAGGGTCCGAGGTCAGAGCCACCTGGGCAATTTTTTTTTTTACTCCA	24
>hsa-miR-519e-3p MIMAT0002829	AAGUGCCUCCUUUUAGAGUGUU	TCGGAAGTGCCTCCTTTTAG	GTGCAGGGTCCGAGGTCAGAGCCACCTGGGCAATTTTTTTTTTTAACACT	25
>hsa-miR-548e-3p MIMAT0005874	AAAAACUGAGACUACUUUUGCA	GTCGGAAAAACTGAGACTACT	GTGCAGGGTCCGAGGTCAGAGCCACCTGGGCAATTTTTTTTTTTGCAAAA	25
>hsa-miR-377-3p MIMAT0000730	AUCACACAAAGGCAACUUUUGU	TCGGATCACACAAAGGCAAC	GTGCAGGGTCCGAGGTCAGAGCCACCTGGGCAATTTTTTTTTTTACAAAA	25
>hsa-miR-3183 MIMAT0015063	GCCUCUCUCGGAGUCGCUCGGA	TGGCCTCTCTCGGAGTCG	GTGCAGGGTCCGAGGTCAGAGCCACCTGGGCAATTTTTTTTTTTCCGAGC	25
>hsa-miR-34a-5p MIMAT0000255	UGGCAGUGUCUUAGCUGGUUGU	CGGTGGCAGTGTCTTAGCT	GTGCAGGGTCCGAGGTCAGAGCCACCTGGGCAATTTTTTTTTTTACAACC	25
>hsa-miR-302a-3p MIMAT0000684	UAAGUGCUUCCAUGUUUUGGUGA	TCGGTAAGTGCTTCCATGTTT	GTGCAGGGTCCGAGGTCAGAGCCACCTGGGCAATTTTTTTTTTTCACCAA	25
>hsa-miR-4328 MIMAT0016926	CCAGUUUUCCCAGGAUU	CGGTCGGCCAGTTTTCCC	GTGCAGGGTCCGAGGTCAGAGCCACCTGGGCAATTTTTTTTTTTAATCCT	25
>hsa-miR-551a MIMAT0003214	GCGACCCACUCUUGGUUUCCA	TGGGCGACCCACTCTTGG	GTGCAGGGTCCGAGGTCAGAGCCACCTGGGCAATTTTTTTTTTTGGAAAC	26
>hsa-miR-345-5p MIMAT0000772	GCUGACUCCUAGUCCAGGGCUC	TGGGCTGACTCCTAGTCCA	GTGCAGGGTCCGAGGTCAGAGCCACCTGGGCAATTTTTTTTTTTGAGCCC	26
>hsa-miR-675-3p MIMAT0006790	CUGUAUGCCCUCACCGCUCA	TTCGGCTGTATGCCCTCAC	GTGCAGGGTCCGAGGTCAGAGCCACCTGGGCAATTTTTTTTTTTGAGCGG	26
>hsa-miR-4464 MIMAT0018988	AAGGUUUGGAUAGAUGCAAUA	TGTCGGAAGGTTTGGATAGAT	GTGCAGGGTCCGAGGTCAGAGCCACCTGGGCAATTTTTTTTTTTATTGCA	26
>hsa-miR-99a-5p MIMAT0000097	AACCCGUAGAUCCGAUCUUGUG	CGGAACCCGTAGATCCGAT	GTGCAGGGTCCGAGGTCAGAGCCACCTGGGCAATTTTTTTTTTTCACAAG	26
>hsa-miR-128-3p MIMAT0000424	UCACAGUGAACCGGUCUCUUU	TCGGTCACAGTGAACCGGT	GTGCAGGGTCCGAGGTCAGAGCCACCTGGGCAATTTTTTTTTTTAAAGAG	26
>hsa-miR-1247-5p MIMAT0005899	ACCCGUCCCGUUCGUCCCCGGA	TGACCCGTCCCGTTCGTC	GTGCAGGGTCCGAGGTCAGAGCCACCTGGGCAATTTTTTTTTTTCCGGGG	26
>hsa-miR-671-3p MIMAT0004819	UCCGGUUCUCAGGGCUCCACC	TGGTCCGGTTCTCAGGGC	GTGCAGGGTCCGAGGTCAGAGCCACCTGGGCAATTTTTTTTTTTGGTGGA	27
>hsa-miR-429 MIMAT0001536	UAAUACUGUCUGGUAAAACCGU	GTCGGTAATACTGTCTGGTAA	GTGCAGGGTCCGAGGTCAGAGCCACCTGGGCAATTTTTTTTTTTACGGTT	27
>hsa-miR-4732-3p MIMAT0019856	GCCCUGACCUGUCCUGUUCUG	TGGGCCCTGACCTGTCCT	GTGCAGGGTCCGAGGTCAGAGCCACCTGGGCAATTTTTTTTTTTCAGAAC	27
>hsa-miR-325 MIMAT0000771	CCUAGUAGGUGUCCAGUAAGUGU	GGCCTAGTAGGTGTCCAGT	GTGCAGGGTCCGAGGTCAGAGCCACCTGGGCAATTTTTTTTTTTACACTT	27
>hsa-miR-380-5p MIMAT0000734	UGGUUGACCAUAGAACAUGCGC	TCGGTGGTTGACCATAGAAC	GTGCAGGGTCCGAGGTCAGAGCCACCTGGGCAATTTTTTTTTTTGCGCAT	27
>hsa-miR-489-3p MIMAT0002805	GUGACAUCACAUAUACGGCAGC	TTCGGGTGACATCACATATAC	GTGCAGGGTCCGAGGTCAGAGCCACCTGGGCAATTTTTTTTTTTGCTGCC	27
>hsa-miR-29c-5p MIMAT0004673	UGACCGAUUUCUCCUGGUGUUC	CGGTGACCGATTTCTCCTG	GTGCAGGGTCCGAGGTCAGAGCCACCTGGGCAATTTTTTTTTTTGAACAC	27
>hsa-miR-146a-5p MIMAT0000449	UGAGAACUGAAUUCCAUGGGUU	TTCGGTGAGAACTGAATTCCA	GTGCAGGGTCCGAGGTCAGAGCCACCTGGGCAATTTTTTTTTTTAACCCA	28
>hsa-miR-448 MIMAT0001532	UUGCAUAUGUAGGAUGUCCCAU	TTCGGTTGCATATGTAGGATG	GTGCAGGGTCCGAGGTCAGAGCCACCTGGGCAATTTTTTTTTTTATGGGA	28
>hsa-miR-433-3p MIMAT0001627	AUCAUGAUGGGCUCCUCGGUGU	CGGATCATGATGGGCTCCT	GTGCAGGGTCCGAGGTCAGAGCCACCTGGGCAATTTTTTTTTTTACACCG	28
>hsa-miR-519d-3p MIMAT0002853	CAAAGUGCCUCCCUUUAGAGUG	CGGCAAAGTGCCTCCCTTT	GTGCAGGGTCCGAGGTCAGAGCCACCTGGGCAATTTTTTTTTTTCACTCT	28
>hsa-miR-326 MIMAT0000756	CCUCUGGGCCCUUCCUCCAG	CGGCCTCTGGGCCCTTC	GTGCAGGGTCCGAGGTCAGAGCCACCTGGGCAATTTTTTTTTTTCTGGAG	28
>hsa-miR-199b-5p MIMAT0000263	CCCAGUGUUUAGACUAUCUGUUC	CGGCCCAGTGTTTAGACTAT	GTGCAGGGTCCGAGGTCAGAGCCACCTGGGCAATTTTTTTTTTTGAACAG	28
>hsa-miR-30b-3p MIMAT0004589	CUGGGAGGUGGAUGUUUACUUC	TGGCTGGGAGGTGGATGTT	GTGCAGGGTCCGAGGTCAGAGCCACCTGGGCAATTTTTTTTTTTGAAGTA	28
>hsa-miR-199a-3p MIMAT0000232	ACAGUAGUCUGCACAUUGGUUA	TCGGACAGTAGTCTGCACAT	GTGCAGGGTCCGAGGTCAGAGCCACCTGGGCAATTTTTTTTTTTAACCAA	29
>hsa-miR-337-3p MIMAT0000754	CUCCUAUAUGAUGCCUUUCUUC	TCGGCTCCTATATGATGCCT	GTGCAGGGTCCGAGGTCAGAGCCACCTGGGCAATTTTTTTTTTTGAAGAA	29
>hsa-miR-1249-3p MIMAT0005901	ACGCCCUUCCCCCCCUUCUUCA	GACGCCCTTCCCCCCC	GTGCAGGGTCCGAGGTCAGAGCCACCTGGGCAATTTTTTTTTTTGAAGAA	29
>hsa-miR-3159 MIMAT0015033	UAGGAUUACAAGUGUCGGCCAC	TTCGGTAGGATTACAAGTGTC	GTGCAGGGTCCGAGGTCAGAGCCACCTGGGCAATTTTTTTTTTTGTGGCC	29
>hsa-miR-9-3p MIMAT0000442	AUAAAGCUAGAUAACCGAAAGU	GTCGGATAAAGCTAGATAACC	GTGCAGGGTCCGAGGTCAGAGCCACCTGGGCAATTTTTTTTTTTACTTTC	29
>hsa-miR-20b-5p MIMAT0001413	CAAAGUGCUCAUAGUGCAGGUAG	TGGCAAAGTGCTCATAGTGC	GTGCAGGGTCCGAGGTCAGAGCCACCTGGGCAATTTTTTTTTTTCTACCT	29
>hsa-miR-152-3p MIMAT0000438	UCAGUGCAUGACAGAACUUGG	TTCGGTCAGTGCATGACAGA	GTGCAGGGTCCGAGGTCAGAGCCACCTGGGCAATTTTTTTTTTTCCAAGT	29
>hsa-miR-548d-5p MIMAT0004812	AAAAGUAAUUGUGGUUUUUGCC	TGTCGGAAAAGTAATTGTGGTT	GTGCAGGGTCCGAGGTCAGAGCCACCTGGGCAATTTTTTTTTTTGGCAAA	30
>hsa-miR-142-5p MIMAT0000433	CAUAAAGUAGAAAGCACUACU	TGTCGGCATAAAGTAGAAAGC	GTGCAGGGTCCGAGGTCAGAGCCACCTGGGCAATTTTTTTTTTTAGTAGT	30
>hsa-miR-20a-5p MIMAT0000075	UAAAGUGCUUAUAGUGCAGGUAG	TCGGTAAAGTGCTTATAGTGC	GTGCAGGGTCCGAGGTCAGAGCCACCTGGGCAATTTTTTTTTTTCTACCT	30
>hsa-miR-507 MIMAT0002879	UUUUGCACCUUUUGGAGUGAA	GTCGGTTTTGCACCTTTTGG	GTGCAGGGTCCGAGGTCAGAGCCACCTGGGCAATTTTTTTTTTTCACTCC	30
>hsa-miR-374b-5p MIMAT0004955	AUAUAAUACAACCUGCUAAGUG	GTCGGATATAATACAACCTGC	GTGCAGGGTCCGAGGTCAGAGCCACCTGGGCAATTTTTTTTTTTCACTTA	30
>hsa-miR-5095 MIMAT0020600	UUACAGGCGUGAACCACCGCG	TCGGTTACAGGCGTGAACC	GTGCAGGGTCCGAGGTCAGAGCCACCTGGGCAATTTTTTTTTTTCGCGGT	30

>hsa-miR-362-3p MIMAT0004683	AACACACCUAUUCAAGGAUUCA	TTCGGAACACACCTATTCAAG	GTGCAGGGTCCGAGGTCAGAGCCACCTGGGCAATTTTTTTTTTTGAATCC	30
>hsa-miR-138-1-3p MIMAT0004607	GCUACUUCACAACACCAGGGCC	CGGGCTACTTCACAACACC	GTGCAGGGTCCGAGGTCAGAGCCACCTGGGCAATTTTTTTTTTTGGCCCT	31
>hsa-miR-532-3p MIMAT0004780	CCUCCCACACCCAAGGCUUGCA	TGCCTCCCACACCCAAGG	GTGCAGGGTCCGAGGTCAGAGCCACCTGGGCAATTTTTTTTTTTGCAAGC	31
>hsa-miR-3191-3p MIMAT0015075	UGGGGACGUAGCUGGCCAGACAG	TTGGGGACGTAGCTGGCC	GTGCAGGGTCCGAGGTCAGAGCCACCTGGGCAATTTTTTTTTTTCTGTCT	31
>hsa-miR-613 MIMAT0003281	AGGAAUGUUCCUUCUUUGCC	TGTCGGAGGAATGTTCCTTC	GTGCAGGGTCCGAGGTCAGAGCCACCTGGGCAATTTTTTTTTTTGGCAAA	31
>hsa-miR-376c-3p MIMAT0000720	AACAUAGAGGAAAUUCCACGU	GGTCGGAACATAGAGGAAATT	GTGCAGGGTCCGAGGTCAGAGCCACCTGGGCAATTTTTTTTTTTACGTGG	31
>hsa-miR-188-5p MIMAT0000457	CAUCCCUUGCAUGGUGGAGGG	TCGGCATCCCTTGCATGGT	GTGCAGGGTCCGAGGTCAGAGCCACCTGGGCAATTTTTTTTTTTCCCTCC	31
>hsa-miR-320a MIMAT0000510	AAAAGCUGGGUUGAGAGGGCGA	TCGGAAAAGCTGGGTTGAGA	GTGCAGGGTCCGAGGTCAGAGCCACCTGGGCAATTTTTTTTTTTCGCCCT	31
>hsa-miR-631 MIMAT0003300	AGACCUGGCCCAGACCUCAGC	TGGAGACCTGGCCCAGAC	GTGCAGGGTCCGAGGTCAGAGCCACCTGGGCAATTTTTTTTTTTGCTGAG	32
>hsa-miR-151a-3p MIMAT0000757	CUAGACUGAAGCUCCUUGAGG	TCGGCTAGACTGAAGCTCC	GTGCAGGGTCCGAGGTCAGAGCCACCTGGGCAATTTTTTTTTTTCCTCAA	32
>hsa-miR-371a-3p MIMAT0000723	AAGUGCCGCCAUCUUUUGAGUGU	TGGAAGTGCCGCCATCTTTT	GTGCAGGGTCCGAGGTCAGAGCCACCTGGGCAATTTTTTTTTTTACACTC	32
>hsa-miR-3923 MIMAT0018198	AACUAGUAAUGUUGGAUUAGGG	GTCGGAACTAGTAATGTTGGA	GTGCAGGGTCCGAGGTCAGAGCCACCTGGGCAATTTTTTTTTTTCCCTAA	32
>hsa-miR-616-3p MIMAT0004805	AGUCAUUGGAGGGUUUGAGCAG	TCGGAGTCATTGGAGGGTTT	GTGCAGGGTCCGAGGTCAGAGCCACCTGGGCAATTTTTTTTTTTCTGCTC	32
>hsa-miR-1208 MIMAT0005873	UCACUGUUCAGACAGGCGGA	TGTCGGTCACTGTTCAGACA	GTGCAGGGTCCGAGGTCAGAGCCACCTGGGCAATTTTTTTTTTTCCGCCT	32
>hsa-miR-22-5p MIMAT0004495	AGUUCUUCAGUGGCAAGCUUUA	TCGGAGTTCTTCAGTGGCAA	GTGCAGGGTCCGAGGTCAGAGCCACCTGGGCAATTTTTTTTTTTAAAGCT	32
>hsa-miR-664a-3p MIMAT0005949	UAUUCAUUUAUCCCCAGCCUACA	TCGGTATTCATTTATCCCCAG	GTGCAGGGTCCGAGGTCAGAGCCACCTGGGCAATTTTTTTTTTTGTAGGC	33
>hsa-miR-483-5p MIMAT0004761	AAGACGGGAGGAAAGAAGGGAG	CGGAAGACGGGAGGAAAGA	GTGCAGGGTCCGAGGTCAGAGCCACCTGGGCAATTTTTTTTTTTCTCCCT	33
>hsa-miR-135a-5p MIMAT0000428	UAUGGCUUUUUAUUCCUAUGUGA	TTCGGTATGGCTTTTTATTCCT	GTGCAGGGTCCGAGGTCAGAGCCACCTGGGCAATTTTTTTTTTTCACATA	33
>hsa-miR-369-3p MIMAT0000721	AAUAAUACAUGGUUGAUCUUU	GGTCGGAATAATACATGGTTG	GTGCAGGGTCCGAGGTCAGAGCCACCTGGGCAATTTTTTTTTTTAAAGAT	33
>hsa-miR-497-5p MIMAT0002820	CAGCAGCACACUGUGGUUUGU	CGGCAGCAGCACACTGTG	GTGCAGGGTCCGAGGTCAGAGCCACCTGGGCAATTTTTTTTTTTACAAAC	33
>hsa-miR-18a-3p MIMAT0002891	ACUGCCCUAAGUGCUCCUUCUGG	TGACTGCCCTAAGTGCTCC	GTGCAGGGTCCGAGGTCAGAGCCACCTGGGCAATTTTTTTTTTTCCAGAA	33
>hsa-miR-1260b MIMAT0015041	AUCCCACCACUGCCACCAU	GTCGGATCCCACCACTGC	GTGCAGGGTCCGAGGTCAGAGCCACCTGGGCAATTTTTTTTTTTATGGTG	33
>hsa-miR-150-5p MIMAT0000451	UCUCCCAACCCUUGUACCAGUG	CGGTCTCCCAACCCTTGTA	GTGCAGGGTCCGAGGTCAGAGCCACCTGGGCAATTTTTTTTTTTCACTGG	34
>hsa-miR-381-3p MIMAT0000736	UAUACAAGGGCAAGCUCUCUGU	TCGGTATACAAGGGCAAGCT	GTGCAGGGTCCGAGGTCAGAGCCACCTGGGCAATTTTTTTTTTTACAGAG	34
>hsa-miR-133b MIMAT0000770	UUUGGUCCCCUUCAACCAGCUA	CGGTTTGGTCCCCTTCAAC	GTGCAGGGTCCGAGGTCAGAGCCACCTGGGCAATTTTTTTTTTTAGCTGG	34
>hsa-miR-15a-3p MIMAT0004488	CAGGCCAUAUUGUGCUGCCUCA	CGGCAGGCCATATTGTGCT	GTGCAGGGTCCGAGGTCAGAGCCACCTGGGCAATTTTTTTTTTTGAGGCA	34
>hsa-miR-26b-5p MIMAT0000083	UUCAAGUAAUUCAGGAUAGGU	TGTCGGTTCAAGTAATTCAGG	GTGCAGGGTCCGAGGTCAGAGCCACCTGGGCAATTTTTTTTTTTACCTAT	34
>hsa-miR-596 MIMAT0003264	AAGCCUGCCCGGCUCCUCGGG	GGAAGCCTGCCCGGCTC	GTGCAGGGTCCGAGGTCAGAGCCACCTGGGCAATTTTTTTTTTTCCCGAG	34
>hsa-miR-1203 MIMAT0005866	CCCGGAGCCAGGAUGCAGCUC	GGCCCGGAGCCAGGATG	GTGCAGGGTCCGAGGTCAGAGCCACCTGGGCAATTTTTTTTTTTGAGCTG	34
>hsa-miR-135b-5p MIMAT0000758	UAUGGCUUUUCAUUCCUAUGUGA	TCGGTATGGCTTTTCATTCCT	GTGCAGGGTCCGAGGTCAGAGCCACCTGGGCAATTTTTTTTTTTCACATA	35
>hsa-miR-181a-5p MIMAT0000256	AACAUUCAACGCUGUCGGUGAGU	TGGAACATTCAACGCTGTCG	GTGCAGGGTCCGAGGTCAGAGCCACCTGGGCAATTTTTTTTTTTACTCAC	35
>hsa-miR-340-5p MIMAT0004692	UUAUAAAGCAAUGAGACUGAUU	TGTCGGTTATAAAGCAATGAGA	GTGCAGGGTCCGAGGTCAGAGCCACCTGGGCAATTTTTTTTTTTAATCAG	35
>hsa-miR-337-5p MIMAT0004695	GAACGGCUUCAUACAGGAGUU	TTCGGGAACGGCTTCATACA	GTGCAGGGTCCGAGGTCAGAGCCACCTGGGCAATTTTTTTTTTTAACTCC	35
>hsa-miR-2355-5p MIMAT0016895	AUCCCCAGAUACAAUGGACAA	GTCGGATCCCCAGATACAAT	GTGCAGGGTCCGAGGTCAGAGCCACCTGGGCAATTTTTTTTTTTGTCCAT	35
>hsa-miR-1205 MIMAT0005869	UCUGCAGGGUUUGCUUUGAG	TTCGGTCTGCAGGGTTTGC	GTGCAGGGTCCGAGGTCAGAGCCACCTGGGCAATTTTTTTTTTTCTCAAA	35
>hsa-miR-140-5p MIMAT0000431	CAGUGGUUUUACCCUAUGGUAG	TCGGCAGTGGTTTTACCCTA	GTGCAGGGTCCGAGGTCAGAGCCACCTGGGCAATTTTTTTTTTTCTACCA	35
>hsa-miR-455-3p MIMAT0004784	GCAGUCCAUGGGCAUAUACAC	CGGGCAGTCCATGGGCAT	GTGCAGGGTCCGAGGTCAGAGCCACCTGGGCAATTTTTTTTTTTGTGTAT	36
>hsa-let-7g-3p MIMAT0004584	CUGUACAGGCCACUGCCUUGC	CGGCTGTACAGGCCACTG	GTGCAGGGTCCGAGGTCAGAGCCACCTGGGCAATTTTTTTTTTTGCAAGG	36
>hsa-miR-147a MIMAT0000251	GUGUGUGGAAAUGCUUCUGC	GTCGGGTGTGTGGAAATGC	GTGCAGGGTCCGAGGTCAGAGCCACCTGGGCAATTTTTTTTTTTGCAGAA	36
>hsa-miR-338-3p MIMAT0000763	UCCAGCAUCAGUGAUUUUGUUG	TCGGTCCAGCATCAGTGATT	GTGCAGGGTCCGAGGTCAGAGCCACCTGGGCAATTTTTTTTTTTCAACAA	36
>hsa-miR-200a-3p MIMAT0000682	UAACACUGUCUGGUAACGAUGU	TTCGGTAACACTGTCTGGTAA	GTGCAGGGTCCGAGGTCAGAGCCACCTGGGCAATTTTTTTTTTTACATCG	36
>hsa-miR-1301-3p MIMAT0005797	UUGCAGCUGCCUGGGAGUGACUUC	TTTGCAGCTGCCTGGGAGT	GTGCAGGGTCCGAGGTCAGAGCCACCTGGGCAATTTTTTTTTTTGAAGTC	36
>hsa-miR-15b-5p MIMAT0000417	UAGCAGCACAUCAUGGUUUACA	CGGTAGCAGCACATCATGG	GTGCAGGGTCCGAGGTCAGAGCCACCTGGGCAATTTTTTTTTTTGTAAAC	36

>hsa-miR-342-5p MIMAT0004694	AGGGGUGCUAUCUGUGAUUGA	TCGGAGGGGTGCTATCTGT	GTGCAGGGTCCGAGGTCAGAGCCACCTGGGCAATTTTTTTTTTTCAATCA	37
>hsa-miR-98-5p MIMAT0000096	UGAGGUAGUAAGUUGUAUUGUU	TTCGGTGAGGTAGTAAGTTGT	GTGCAGGGTCCGAGGTCAGAGCCACCTGGGCAATTTTTTTTTTTAACAAT	37
>hsa-miR-30c-5p MIMAT0000244	UGUAAACAUCCUACACUCUCAGC	TCGGTGTAAACATCCTACACT	GTGCAGGGTCCGAGGTCAGAGCCACCTGGGCAATTTTTTTTTTTGCTGAG	37
>hsa-miR-4516 MIMAT0019053	GGGAGAAGGGUCGGGGC	TGGTCGGGGGAGAAGGGT	GTGCAGGGTCCGAGGTCAGAGCCACCTGGGCAATTTTTTTTTTTGCCCCG	37
>hsa-miR-22-3p MIMAT0000077	AAGCUGCCAGUUGAAGAACUGU	CGGAAGCTGCCAGTTGAAG	GTGCAGGGTCCGAGGTCAGAGCCACCTGGGCAATTTTTTTTTTTACAGTT	37
>hsa-miR-103a-2-5p MIMAT0009196	AGCUUCUUUACAGUGCUGCCUUG	CGGAGCTTCTTTACAGTGCT	GTGCAGGGTCCGAGGTCAGAGCCACCTGGGCAATTTTTTTTTTTCAAGGC	37
>hsa-miR-636 MIMAT0003306	UGUGCUUGCUCGUCCCGCCCGCA	GTGTGCTTGCTCGTCCCG	GTGCAGGGTCCGAGGTCAGAGCCACCTGGGCAATTTTTTTTTTTGCGGGC	37
>hsa-miR-186-5p MIMAT0000456	CAAAGAAUUCUCCUUUUGGGCU	GTCGGCAAAGAATTCTCCTTT	GTGCAGGGTCCGAGGTCAGAGCCACCTGGGCAATTTTTTTTTTTAGCCCA	38
>hsa-miR-127-5p MIMAT0004604	CUGAAGCUCAGAGGGCUCUGAU	GGCTGAAGCTCAGAGGGC	GTGCAGGGTCCGAGGTCAGAGCCACCTGGGCAATTTTTTTTTTTATCAGA	38
>hsa-miR-197-3p MIMAT0000227	UUCACCACCUUCUCCACCCAGC	CGGTTCACCACCTTCTCCA	GTGCAGGGTCCGAGGTCAGAGCCACCTGGGCAATTTTTTTTTTTGCTGGG	38
>hsa-miR-203a-3p MIMAT0000264	GUGAAAUGUUUAGGACCACUAG	TTCGGGTGAAATGTTTAGGAC	GTGCAGGGTCCGAGGTCAGAGCCACCTGGGCAATTTTTTTTTTTCTAGTG	38
>hsa-miR-30e-3p MIMAT0000693	CUUUCAGUCGGAUGUUUACAGC	TCGGCTTTCAGTCGGATGTT	GTGCAGGGTCCGAGGTCAGAGCCACCTGGGCAATTTTTTTTTTTGCTGTA	38
>hsa-miR-17-3p MIMAT0000071	ACUGCAGUGAAGGCACUUGUAG	TGGACTGCAGTGAAGGCAC	GTGCAGGGTCCGAGGTCAGAGCCACCTGGGCAATTTTTTTTTTTCTACAA	38
>hsa-miR-409-3p MIMAT0001639	GAAUGUUGCUCGGUGAACCCCU	CGGGAATGTTGCTCGGTGA	GTGCAGGGTCCGAGGTCAGAGCCACCTGGGCAATTTTTTTTTTTAGGGGT	38
>hsa-miR-139-5p MIMAT0000250	UCUACAGUGCACGUGUCUCCAGU	GGTCTACAGTGCACGTGTC	GTGCAGGGTCCGAGGTCAGAGCCACCTGGGCAATTTTTTTTTTTACTGGA	39
>hsa-miR-299-3p MIMAT0000687	UAUGUGGGAUGGUAAACCGCUU	TTCGGTATGTGGGATGGTAAA	GTGCAGGGTCCGAGGTCAGAGCCACCTGGGCAATTTTTTTTTTTAAGCGG	39
>hsa-miR-509-5p MIMAT0004779	UACUGCAGACAGUGGCAAUCA	TCGGTACTGCAGACAGTGG	GTGCAGGGTCCGAGGTCAGAGCCACCTGGGCAATTTTTTTTTTTGATTGC	39
>hsa-miR-320b MIMAT0005792	AAAAGCUGGGUUGAGAGGGCAA	TCGGAAAAGCTGGGTTGAGA	GTGCAGGGTCCGAGGTCAGAGCCACCTGGGCAATTTTTTTTTTTGCCCTC	39
>hsa-miR-425-5p MIMAT0003393	AAUGACACGAUCACUCCCGUUGA	TGGAATGACACGATCACTCC	GTGCAGGGTCCGAGGTCAGAGCCACCTGGGCAATTTTTTTTTTTCAACGG	39
>hsa-miR-96-5p MIMAT0000095	UUUGGCACUAGCACAUUUUUGCU	CGGTTTGGCACTAGCACATT	GTGCAGGGTCCGAGGTCAGAGCCACCTGGGCAATTTTTTTTTTTAGCAAA	39
>hsa-miR-124-3p MIMAT0000422	UAAGGCACGCGGUGAAUGCC	TCGGTAAGGCACGCGGTG	GTGCAGGGTCCGAGGTCAGAGCCACCTGGGCAATTTTTTTTTTTGGCATT	39
>hsa-miR-136-5p MIMAT0000448	ACUCCAUUUGUUUUGAUGAUGGA	TTCGGACTCCATTTGTTTTGAT	GTGCAGGGTCCGAGGTCAGAGCCACCTGGGCAATTTTTTTTTTTCCATCA	40
>hsa-miR-146b-5p MIMAT0002809	UGAGAACUGAAUUCCAUAGGCU	TTCGGTGAGAACTGAATTCCA	GTGCAGGGTCCGAGGTCAGAGCCACCTGGGCAATTTTTTTTTTTAGCCTA	40
>hsa-miR-4770 MIMAT0019924	UGAGAUGACACUGUAGCU	CGGTCGGTGAGATGACACT	GTGCAGGGTCCGAGGTCAGAGCCACCTGGGCAATTTTTTTTTTTAGCTAC	40
>hsa-miR-590-3p MIMAT0004801	UAAUUUUAUGUAUAAGCUAGU	TCGGTCGGTAATTTTATGTATAA	GTGCAGGGTCCGAGGTCAGAGCCACCTGGGCAATTTTTTTTTTTACTAGC	40
>hsa-miR-324-5p MIMAT0000761	CGCAUCCCCUAGGGCAUUGGUGU	GCGCATCCCCTAGGGCAT	GTGCAGGGTCCGAGGTCAGAGCCACCTGGGCAATTTTTTTTTTTACACCA	40
>hsa-miR-30c-2-3p MIMAT0004550	CUGGGAGAAGGCUGUUUACUCU	TGGCTGGGAGAAGGCTGTT	GTGCAGGGTCCGAGGTCAGAGCCACCTGGGCAATTTTTTTTTTTAGAGTA	40
>hsa-miR-181c-3p MIMAT0004559	AACCAUCGACCGUUGAGUGGAC	CGGAACCATCGACCGTTGA	GTGCAGGGTCCGAGGTCAGAGCCACCTGGGCAATTTTTTTTTTTGTCCAC	40
>hsa-miR-16-2-3p MIMAT0004518	CCAAUAUUACUGUGCUGCUUUA	TTCGGCCAATATTACTGTGCT	GTGCAGGGTCCGAGGTCAGAGCCACCTGGGCAATTTTTTTTTTTAAAGCA	41
>hsa-miR-130b-3p MIMAT0000691	CAGUGCAAUGAUGAAAGGGCAU	TTCGGCAGTGCAATGATGAAA	GTGCAGGGTCCGAGGTCAGAGCCACCTGGGCAATTTTTTTTTTTATGCCC	41
>hsa-miR-215-5p MIMAT0000272	AUGACCUAUGAAUUGACAGAC	TGTCGGATGACCTATGAATTG	GTGCAGGGTCCGAGGTCAGAGCCACCTGGGCAATTTTTTTTTTTGTCTGT	41
>hsa-miR-766-3p MIMAT0003888	ACUCCAGCCCCACAGCCUCAGC	TGACTCCAGCCCCACAGC	GTGCAGGGTCCGAGGTCAGAGCCACCTGGGCAATTTTTTTTTTTGCTGAG	41
>hsa-miR-141-3p MIMAT0000432	UAACACUGUCUGGUAAAGAUGG	TTCGGTAACACTGTCTGGTAA	GTGCAGGGTCCGAGGTCAGAGCCACCTGGGCAATTTTTTTTTTTCCATCT	41
>hsa-miR-33a-5p MIMAT0000091	GUGCAUUGUAGUUGCAUUGCA	TTCGGGTGCATTGTAGTTGC	GTGCAGGGTCCGAGGTCAGAGCCACCTGGGCAATTTTTTTTTTTGCAATG	41
>hsa-miR-1-3p MIMAT0000416	UGGAAUGUAAAGAAGUAUGUAU	GTCGGTGGAATGTAAAGAAGT	GTGCAGGGTCCGAGGTCAGAGCCACCTGGGCAATTTTTTTTTTTATACAT	41
>hsa-miR-30d-3p MIMAT0004551	CUUUCAGUCAGAUGUUUGCUGC	TTCGGCTTTCAGTCAGATGTT	GTGCAGGGTCCGAGGTCAGAGCCACCTGGGCAATTTTTTTTTTTGCAGCA	42
>hsa-miR-191-5p MIMAT0000440	CAACGGAAUCCCAAAAGCAGCUG	TGGCAACGGAATCCCAAAAG	GTGCAGGGTCCGAGGTCAGAGCCACCTGGGCAATTTTTTTTTTTCAGCTG	42
>hsa-miR-129-5p MIMAT0000242	CUUUUUGCGGUCUGGGCUUGC	TCGGCTTTTTGCGGTCTGG	GTGCAGGGTCCGAGGTCAGAGCCACCTGGGCAATTTTTTTTTTTGCAAGC	42
>hsa-miR-4538 MIMAT0019081	GAGCUUGGAUGAGCUGGGCUGA	TGGGAGCTTGGATGAGCTG	GTGCAGGGTCCGAGGTCAGAGCCACCTGGGCAATTTTTTTTTTTCAGCCC	42
>hsa-miR-29a-3p MIMAT0000086	UAGCACCAUCUGAAAUCGGUUA	TTCGGTAGCACCATCTGAAAT	GTGCAGGGTCCGAGGTCAGAGCCACCTGGGCAATTTTTTTTTTTAACCGA	42
>hsa-miR-942-5p MIMAT0004985	UCUUCUCUGUUUUGGCCAUGUG	TCGGTCTTCTCTGTTTTGGC	GTGCAGGGTCCGAGGTCAGAGCCACCTGGGCAATTTTTTTTTTTCACATG	42
>hsa-miR-93-5p MIMAT0000093	CAAAGUGCUGUUCGUGCAGGUAG	GGCAAAGTGCTGTTCGTGC	GTGCAGGGTCCGAGGTCAGAGCCACCTGGGCAATTTTTTTTTTTCTACCT	42
>hsa-miR-342-3p MIMAT0000753	UCUCACACAGAAAUCGCACCCGU	TGGTCTCACACAGAAATCGC	GTGCAGGGTCCGAGGTCAGAGCCACCTGGGCAATTTTTTTTTTTACGGGT	43

>hsa-miR-487b-3p MIMAT0003180	AAUCGUACAGGGUCAUCCACUU	TCGGAATCGTACAGGGTCAT	GTGCAGGGTCCGAGGTCAGAGCCACCTGGGCAATTTTTTTTTTTAAGTGG	43
>hsa-miR-122-5p MIMAT0000421	UGGAGUGUGACAAUGGUGUUUG	CGGTGGAGTGTGACAATGG	GTGCAGGGTCCGAGGTCAGAGCCACCTGGGCAATTTTTTTTTTTCAAACA	43
>hsa-miR-301a-3p MIMAT0000688	CAGUGCAAUAGUAUUGUCAAAGC	TCGGCAGTGCAATAGTATTGT	GTGCAGGGTCCGAGGTCAGAGCCACCTGGGCAATTTTTTTTTTTGCTTTG	43
>hsa-miR-4524a-3p MIMAT0019063	UGAGACAGGCUUAUGCUGCUAU	CGGTGAGACAGGCTTATGC	GTGCAGGGTCCGAGGTCAGAGCCACCTGGGCAATTTTTTTTTTTATAGCA	43
>hsa-miR-320e MIMAT0015072	AAAGCUGGGUUGAGAAGG	TGGTCGGAAAGCTGGGTTG	GTGCAGGGTCCGAGGTCAGAGCCACCTGGGCAATTTTTTTTTTTCCTTCT	43
>hsa-miR-26a-5p MIMAT0000082	UUCAAGUAAUCCAGGAUAGGCU	TTCGGTTCAAGTAATCCAGGA	GTGCAGGGTCCGAGGTCAGAGCCACCTGGGCAATTTTTTTTTTTAGCCTA	43
>hsa-miR-492 MIMAT0002812	AGGACCUGCGGGACAAGAUUCUU	GAGGACCTGCGGGACAAG	GTGCAGGGTCCGAGGTCAGAGCCACCTGGGCAATTTTTTTTTTTAAGAAT	44
>hsa-let-7f-1-3p MIMAT0004486	CUAUACAAUCUAUUGCCUUCCC	GTCGGCTATACAATCTATTGC	GTGCAGGGTCCGAGGTCAGAGCCACCTGGGCAATTTTTTTTTTTGGGAAG	44
>hsa-miR-200c-3p MIMAT0000617	UAAUACUGCCGGGUAAUGAUGGA	CGGTAATACTGCCGGGTAAT	GTGCAGGGTCCGAGGTCAGAGCCACCTGGGCAATTTTTTTTTTTCCATCA	44
>hsa-miR-378h MIMAT0018984	ACUGGACUUGGUGUCAGAUGG	TCGGACTGGACTTGGTGTC	GTGCAGGGTCCGAGGTCAGAGCCACCTGGGCAATTTTTTTTTTTCCATCT	44
>hsa-miR-885-5p MIMAT0004947	UCCAUUACACUACCCUGCCUCU	TCGGTCCATTACACTACCCT	GTGCAGGGTCCGAGGTCAGAGCCACCTGGGCAATTTTTTTTTTTAGAGGC	44
>hsa-miR-153-3p MIMAT0000439	UUGCAUAGUCACAAAAGUGAUC	GTCGGTTGCATAGTCACAAAA	GTGCAGGGTCCGAGGTCAGAGCCACCTGGGCAATTTTTTTTTTTGATCAC	44
>hsa-miR-3655 MIMAT0018075	GCUUGUCGCUGCGGUGUUGCU	TGGGCTTGTCGCTGCGGT	GTGCAGGGTCCGAGGTCAGAGCCACCTGGGCAATTTTTTTTTTTAGCAAC	44
>hsa-miR-134-5p MIMAT0000447	UGUGACUGGUUGACCAGAGGGG	CGGTGTGACTGGTTGACCA	GTGCAGGGTCCGAGGTCAGAGCCACCTGGGCAATTTTTTTTTTTCCCCTC	45
>hsa-let-7e-5p MIMAT0000066	UGAGGUAGGAGGUUGUAUAGUU	CGGTGAGGTAGGAGGTTGT	GTGCAGGGTCCGAGGTCAGAGCCACCTGGGCAATTTTTTTTTTTAACTAT	45
>hsa-miR-145-5p MIMAT0000437	GUCCAGUUUUCCCAGGAAUCCCU	GGGTCCAGTTTTCCCAGGA	GTGCAGGGTCCGAGGTCAGAGCCACCTGGGCAATTTTTTTTTTTAGGGAT	45
>hsa-miR-331-3p MIMAT0000760	GCCCCUGGGCCUAUCCUAGAA	GGGCCCCTGGGCCTATC	GTGCAGGGTCCGAGGTCAGAGCCACCTGGGCAATTTTTTTTTTTCTAGGA	45
>hsa-miR-196b-5p MIMAT0001080	UAGGUAGUUUCCUGUUGUUGGG	TTCGGTAGGTAGTTTCCTGTT	GTGCAGGGTCCGAGGTCAGAGCCACCTGGGCAATTTTTTTTTTTCCCAAC	45
>hsa-miR-23a-3p MIMAT0000078	AUCACAUUGCCAGGGAUUUCC	TCGGATCACATTGCCAGGG	GTGCAGGGTCCGAGGTCAGAGCCACCTGGGCAATTTTTTTTTTTGGAAAT	45
>hsa-miR-125b-1-3p MIMAT0004592	ACGGGUUAGGCUCUUGGGAGCU	TGGACGGGTTAGGCTCTTG	GTGCAGGGTCCGAGGTCAGAGCCACCTGGGCAATTTTTTTTTTTAGCTCC	45
>hsa-miR-147b MIMAT0004928	GUGUGCGGAAAUGCUUCUGCUA	CGGGTGTGCGGAAATGCTT	GTGCAGGGTCCGAGGTCAGAGCCACCTGGGCAATTTTTTTTTTTAGCAGA	46
>hsa-miR-27a-5p MIMAT0004501	AGGGCUUAGCUGCUUGUGAGCA	TGGAGGGCTTAGCTGCTTG	GTGCAGGGTCCGAGGTCAGAGCCACCTGGGCAATTTTTTTTTTTGCTCAC	46
>hsa-miR-3131 MIMAT0014996	UCGAGGACUGGUGGAAGGGCCUU	TGTCGAGGACTGGTGGAAG	GTGCAGGGTCCGAGGTCAGAGCCACCTGGGCAATTTTTTTTTTTAAGGCC	46
>hsa-miR-133a-3p MIMAT0000427	UUUGGUCCCCUUCAACCAGCUG	CGGTTTGGTCCCCTTCAAC	GTGCAGGGTCCGAGGTCAGAGCCACCTGGGCAATTTTTTTTTTTCAGCTG	46
>hsa-miR-3907 MIMAT0018179	AGGUGCUCCAGGCUGGCUCACA	TGAGGTGCTCCAGGCTGG	GTGCAGGGTCCGAGGTCAGAGCCACCTGGGCAATTTTTTTTTTTGTGAGC	46
>hsa-miR-484 MIMAT0002174	UCAGGCUCAGUCCCCUCCCGAU	GGTCAGGCTCAGTCCCCT	GTGCAGGGTCCGAGGTCAGAGCCACCTGGGCAATTTTTTTTTTTATCGGG	46
>hsa-miR-3129-5p MIMAT0014992	GCAGUAGUGUAGAGAUUGGUUU	TCGGGCAGTAGTGTAGAGAT	GTGCAGGGTCCGAGGTCAGAGCCACCTGGGCAATTTTTTTTTTTAAACCA	46
>hsa-miR-144-3p MIMAT0000436	UACAGUAUAGAUGAUGUACU	TGGTCGGTACAGTATAGATGA	GTGCAGGGTCCGAGGTCAGAGCCACCTGGGCAATTTTTTTTTTTAGTACA	47
>hsa-miR-520e MIMAT0002825	AAAGUGCUUCCUUUUUGAGGG	TGTCGGAAAGTGCTTCCTTTT	GTGCAGGGTCCGAGGTCAGAGCCACCTGGGCAATTTTTTTTTTTCCCTCA	47
>hsa-miR-210-3p MIMAT0000267	CUGUGCGUGUGACAGCGGCUGA	GGCTGTGCGTGTGACAGC	GTGCAGGGTCCGAGGTCAGAGCCACCTGGGCAATTTTTTTTTTTCAGCCG	47
>hsa-miR-494-3p MIMAT0002816	UGAAACAUACACGGGAAACCUC	TCGGTGAAACATACACGGGA	GTGCAGGGTCCGAGGTCAGAGCCACCTGGGCAATTTTTTTTTTTGAGGTT	47
>hsa-miR-2110 MIMAT0010133	UUGGGGAAACGGCCGCUGAGUG	TGTTGGGGAAACGGCCGC	GTGCAGGGTCCGAGGTCAGAGCCACCTGGGCAATTTTTTTTTTTCACTCA	47
>hsa-miR-1207-3p MIMAT0005872	UCAGCUGGCCCUCAUUUC	TGTCGGTCAGCTGGCCCT	GTGCAGGGTCCGAGGTCAGAGCCACCTGGGCAATTTTTTTTTTTGAAATG	47
>hsa-let-7a-3p MIMAT0004481	CUAUACAAUCUACUGUCUUUC	TGTCGGCTATACAATCTACTG	GTGCAGGGTCCGAGGTCAGAGCCACCTGGGCAATTTTTTTTTTTGAAAGA	47
>hsa-miR-31-5p MIMAT0000089	AGGCAAGAUGCUGGCAUAGCU	CGGAGGCAAGATGCTGGC	GTGCAGGGTCCGAGGTCAGAGCCACCTGGGCAATTTTTTTTTTTAGCTAT	48
>hsa-miR-522-3p MIMAT0002868	AAAAUGGUUCCCUUUAGAGUGU	GTCGGAAAATGGTTCCCTTTA	GTGCAGGGTCCGAGGTCAGAGCCACCTGGGCAATTTTTTTTTTTACACTC	48
>hsa-miR-188-3p MIMAT0004613	CUCCCACAUGCAGGGUUUGCA	TGGCTCCCACATGCAGGG	GTGCAGGGTCCGAGGTCAGAGCCACCTGGGCAATTTTTTTTTTTGCAAAC	48
>hsa-miR-425-3p MIMAT0001343	AUCGGGAAUGUCGUGUCCGCCC	CGGATCGGGAATGTCGTGT	GTGCAGGGTCCGAGGTCAGAGCCACCTGGGCAATTTTTTTTTTTGGGCGG	48
>hsa-miR-449a MIMAT0001541	UGGCAGUGUAUUGUUAGCUGGU	TTCGGTGGCAGTGTATTGTTA	GTGCAGGGTCCGAGGTCAGAGCCACCTGGGCAATTTTTTTTTTTACCAGC	48
>hsa-miR-1204 MIMAT0005868	UCGUGGCCUGGUCUCCAUUAU	TGGTCGTGGCCTGGTCTC	GTGCAGGGTCCGAGGTCAGAGCCACCTGGGCAATTTTTTTTTTTATAATG	48
>hsa-miR-137 MIMAT0000429	UUAUUGCUUAAGAAUACGCGUAG	GTCGGTTATTGCTTAAGAATAC	GTGCAGGGTCCGAGGTCAGAGCCACCTGGGCAATTTTTTTTTTTCTACGC	48
>hsa-miR-3911 MIMAT0018185	UGUGUGGAUCCUGGAGGAGGCA	TGGTGTGTGGATCCTGGAG	GTGCAGGGTCCGAGGTCAGAGCCACCTGGGCAATTTTTTTTTTTGCCTCC	49
>hsa-miR-487a-3p MIMAT0002178	AAUCAUACAGGGACAUCCAGUU	TTCGGAATCATACAGGGACAT	GTGCAGGGTCCGAGGTCAGAGCCACCTGGGCAATTTTTTTTTTTAACTGG	49

>hsa-miR-3200-3p MIMAT0015085	CACCUUGCGCUACUCAGGUCUG	TGGCACCTTGCGCTACTCA	GTGCAGGGTCCGAGGTCAGAGCCACCTGGGCAATTTTTTTTTTTCAGACC	49
>hsa-miR-501-5p MIMAT0002872	AAUCCUUUGUCCCUGGGUGAGA	CGGAATCCTTTGTCCCTGG	GTGCAGGGTCCGAGGTCAGAGCCACCTGGGCAATTTTTTTTTTTCTCACC	49
>hsa-miR-183-3p MIMAT0004560	GUGAAUUACCGAAGGGCCAUAA	CGGGTGAATTACCGAAGGG	GTGCAGGGTCCGAGGTCAGAGCCACCTGGGCAATTTTTTTTTTTATGGCC	49
>hsa-miR-194-5p MIMAT0000460	UGUAACAGCAACUCCAUGUGGA	TCGGTGTAACAGCAACTCCA	GTGCAGGGTCCGAGGTCAGAGCCACCTGGGCAATTTTTTTTTTTCCACAT	49
>hsa-miR-4267 MIMAT0016893	UCCAGCUCGGUGGCAC	CGGTCGGTCCAGCTCGG	GTGCAGGGTCCGAGGTCAGAGCCACCTGGGCAATTTTTTTTTTTGTGCCA	49
>hsa-miR-15b-3p MIMAT0004586	CGAAUCAUUAUUUGCUGCUCUA	GTCGGCGAATCATTATTTGCT	GTGCAGGGTCCGAGGTCAGAGCCACCTGGGCAATTTTTTTTTTTAGAGCA	50
>hsa-miR-222-3p MIMAT0000279	AGCUACAUCUGGCUACUGGGU	TTCGGAGCTACATCTGGCTA	GTGCAGGGTCCGAGGTCAGAGCCACCTGGGCAATTTTTTTTTTTACCCAG	50
>hsa-miR-3610 MIMAT0017987	GAAUCGGAAAGGAGGCGCCG	GTCGGGAATCGGAAAGGAG	GTGCAGGGTCCGAGGTCAGAGCCACCTGGGCAATTTTTTTTTTTCGGCGC	50
>hsa-let-7i-5p MIMAT0000415	UGAGGUAGUAGUUUGUGCUGUU	TTCGGTGAGGTAGTAGTTTGT	GTGCAGGGTCCGAGGTCAGAGCCACCTGGGCAATTTTTTTTTTTAACAGC	50
>hsa-miR-4732-5p MIMAT0019855	UGUAGAGCAGGGAGCAGGAAGCU	TGTGTAGAGCAGGGAGCAG	GTGCAGGGTCCGAGGTCAGAGCCACCTGGGCAATTTTTTTTTTTAGCTTC	50
>hsa-miR-17-5p MIMAT0000070	CAAAGUGCUUACAGUGCAGGUAG	TGGCAAAGTGCTTACAGTGC	GTGCAGGGTCCGAGGTCAGAGCCACCTGGGCAATTTTTTTTTTTCTACCT	50
>hsa-miR-378g MIMAT0018937	ACUGGGCUUGGAGUCAGAAG	TTCGGACTGGGCTTGGAGT	GTGCAGGGTCCGAGGTCAGAGCCACCTGGGCAATTTTTTTTTTTCTTCTG	50
>hsa-miR-324-3p MIMAT0000762	ACUGCCCCAGGUGCUGCUGG	CGGACTGCCCCAGGTGC	GTGCAGGGTCCGAGGTCAGAGCCACCTGGGCAATTTTTTTTTTTCCAGCA	51
>hsa-miR-4274 MIMAT0016906	CAGCAGUCCCUCCCCCUG	TGTCGGCAGCAGTCCCTC	GTGCAGGGTCCGAGGTCAGAGCCACCTGGGCAATTTTTTTTTTTCAGGGG	51
>hsa-miR-516a-5p MIMAT0004770	UUCUCGAGGAAAGAAGCACUUUC	TGGTTCTCGAGGAAAGAAGC	GTGCAGGGTCCGAGGTCAGAGCCACCTGGGCAATTTTTTTTTTTGAAAGT	51
>hsa-miR-605-5p MIMAT0003273	UAAAUCCCAUGGUGCCUUCUCCU	TGGTAAATCCCATGGTGCCT	GTGCAGGGTCCGAGGTCAGAGCCACCTGGGCAATTTTTTTTTTTAGGAGA	51
>hsa-miR-432-5p MIMAT0002814	UCUUGGAGUAGGUCAUUGGGUGG	CGGTCTTGGAGTAGGTCATT	GTGCAGGGTCCGAGGTCAGAGCCACCTGGGCAATTTTTTTTTTTCCACCC	51
>hsa-miR-4802-3p MIMAT0019982	UACAUGGAUGGAAACCUUCAAGC	CGGTACATGGATGGAAACCT	GTGCAGGGTCCGAGGTCAGAGCCACCTGGGCAATTTTTTTTTTTGCTTGA	51
>hsa-miR-27b-3p MIMAT0000419	UUCACAGUGGCUAAGUUCUGC	TTCGGTTCACAGTGGCTAAG	GTGCAGGGTCCGAGGTCAGAGCCACCTGGGCAATTTTTTTTTTTGCAGAA	51
>hsa-miR-5000-3p MIMAT0021020	UCAGGACACUUCUGAACUUGGA	TCGGTCAGGACACTTCTGAA	GTGCAGGGTCCGAGGTCAGAGCCACCTGGGCAATTTTTTTTTTTCCAAGT	52
>hsa-miR-493-3p MIMAT0003161	UGAAGGUCUACUGUGUGCCAGG	TCGGTGAAGGTCTACTGTGT	GTGCAGGGTCCGAGGTCAGAGCCACCTGGGCAATTTTTTTTTTTCCTGGC	52
>hsa-let-7g-5p MIMAT0000414	UGAGGUAGUAGUUUGUACAGUU	TTCGGTGAGGTAGTAGTTTGT	GTGCAGGGTCCGAGGTCAGAGCCACCTGGGCAATTTTTTTTTTTAACTGT	52
>hsa-miR-412-3p MIMAT0002170	ACUUCACCUGGUCCACUAGCCGU	GGACTTCACCTGGTCCACT	GTGCAGGGTCCGAGGTCAGAGCCACCTGGGCAATTTTTTTTTTTACGGCT	52
>hsa-miR-1539 MIMAT0007401	UCCUGCGCGUCCCAGAUGCCC	GGTCCTGCGCGTCCCAG	GTGCAGGGTCCGAGGTCAGAGCCACCTGGGCAATTTTTTTTTTTGGGCAT	52
>hsa-miR-3666 MIMAT0018088	CAGUGCAAGUGUAGAUGCCGA	TTCGGCAGTGCAAGTGTAGA	GTGCAGGGTCCGAGGTCAGAGCCACCTGGGCAATTTTTTTTTTTCGGCAT	52
>hsa-miR-192-5p MIMAT0000222	CUGACCUAUGAAUUGACAGCC	GTCGGCTGACCTATGAATTG	GTGCAGGGTCCGAGGTCAGAGCCACCTGGGCAATTTTTTTTTTTGGCTGT	52
>hsa-miR-3613-3p MIMAT0017991	ACAAAAAAAAAAGCCCAACCCUUC	TCGGACAAAAAAAAAAGCCCAA	GTGCAGGGTCCGAGGTCAGAGCCACCTGGGCAATTTTTTTTTTTGAAGGG	53
>hsa-miR-376a-3p MIMAT0000729	AUCAUAGAGGAAAAUCCACGU	GGTCGGATCATAGAGGAAAAT	GTGCAGGGTCCGAGGTCAGAGCCACCTGGGCAATTTTTTTTTTTACGTGG	53
>hsa-miR-574-5p MIMAT0004795	UGAGUGUGUGUGUGUGAGUGUGU	TGGTGAGTGTGTGTGTGTGA	GTGCAGGGTCCGAGGTCAGAGCCACCTGGGCAATTTTTTTTTTTACACAC	53
>hsa-let-7i-3p MIMAT0004585	CUGCGCAAGCUACUGCCUUGCU	GGCTGCGCAAGCTACTGC	GTGCAGGGTCCGAGGTCAGAGCCACCTGGGCAATTTTTTTTTTTAGCAAG	53
>hsa-miR-4422 MIMAT0018935	AAAAGCAUCAGGAAGUACCCA	GTCGGAAAAGCATCAGGAAG	GTGCAGGGTCCGAGGTCAGAGCCACCTGGGCAATTTTTTTTTTTGGGTAC	53
>hsa-miR-7-5p MIMAT0000252	UGGAAGACUAGUGAUUUUGUUGU	TCGGTGGAAGACTAGTGATTT	GTGCAGGGTCCGAGGTCAGAGCCACCTGGGCAATTTTTTTTTTTACAACA	53
>hsa-miR-101-3p MIMAT0000099	UACAGUACUGUGAUAACUGAA	TGTCGGTACAGTACTGTGATA	GTGCAGGGTCCGAGGTCAGAGCCACCTGGGCAATTTTTTTTTTTCAGTTA	53
>hsa-miR-23b-3p MIMAT0000418	AUCACAUUGCCAGGGAUUACC	TCGGATCACATTGCCAGGG	GTGCAGGGTCCGAGGTCAGAGCCACCTGGGCAATTTTTTTTTTTGGTAAT	54
>hsa-miR-223-3p MIMAT0000280	UGUCAGUUUGUCAAAUACCCCA	GTCGGTGTCAGTTTGTCAAAT	GTGCAGGGTCCGAGGTCAGAGCCACCTGGGCAATTTTTTTTTTTGGGGTA	54
>hsa-miR-183-5p MIMAT0000261	UAUGGCACUGGUAGAAUUCACU	TCGGTATGGCACTGGTAGAA	GTGCAGGGTCCGAGGTCAGAGCCACCTGGGCAATTTTTTTTTTTAGTGAA	54
>hsa-miR-1281 MIMAT0005939	UCGCCUCCUCCUCUCCC	GGTCGGTCGCCTCCTCC	GTGCAGGGTCCGAGGTCAGAGCCACCTGGGCAATTTTTTTTTTTGGGAGA	54
>hsa-miR-574-3p MIMAT0003239	CACGCUCAUGCACACACCCACA	TGGCACGCTCATGCACACA	GTGCAGGGTCCGAGGTCAGAGCCACCTGGGCAATTTTTTTTTTTGTGGGT	54
>hsa-miR-30a-5p MIMAT0000087	UGUAAACAUCCUCGACUGGAAG	TCGGTGTAAACATCCTCGAC	GTGCAGGGTCCGAGGTCAGAGCCACCTGGGCAATTTTTTTTTTTCTTCCA	54
>hsa-miR-191-3p MIMAT0001618	GCUGCGCUUGGAUUUCGUCCCC	TGGGCTGCGCTTGGATTTC	GTGCAGGGTCCGAGGTCAGAGCCACCTGGGCAATTTTTTTTTTTGGGGAC	54
>hsa-miR-193a-3p MIMAT0000459	AACUGGCCUACAAAGUCCCAGU	TCGGAACTGGCCTACAAAGT	GTGCAGGGTCCGAGGTCAGAGCCACCTGGGCAATTTTTTTTTTTACTGGG	55
>hsa-miR-576-5p MIMAT0003241	AUUCUAAUUUCUCCACGUCUUU	GTCGGATTCTAATTTCTCCAC	GTGCAGGGTCCGAGGTCAGAGCCACCTGGGCAATTTTTTTTTTTAAAGAC	55
>hsa-miR-1910-5p MIMAT0007884	CCAGUCCUGUGCCUGCCGCCU	TGGCCAGTCCTGTGCCTG	GTGCAGGGTCCGAGGTCAGAGCCACCTGGGCAATTTTTTTTTTTAGGCGG	55

>hsa-miR-542-5p MIMAT0003340	UCGGGGAUCAUCAUGUCACGAGA	GGTCGGGGATCATCATGTC	GTGCAGGGTCCGAGGTCAGAGCCACCTGGGCAATTTTTTTTTTTCTCGTG	55
>hsa-miR-454-3p MIMAT0003885	UAGUGCAAUAUUGCUUAUAGGGU	TTCGGTAGTGCAATATTGCTTA	GTGCAGGGTCCGAGGTCAGAGCCACCTGGGCAATTTTTTTTTTTACCCTA	55
>hsa-miR-218-5p MIMAT0000275	UUGUGCUUGAUCUAACCAUGU	TGTCGGTTGTGCTTGATCTAA	GTGCAGGGTCCGAGGTCAGAGCCACCTGGGCAATTTTTTTTTTTACATGG	55
>hsa-miR-671-5p MIMAT0003880	AGGAAGCCCUGGAGGGGCUGGAG	TAGGAAGCCCTGGAGGGG	GTGCAGGGTCCGAGGTCAGAGCCACCTGGGCAATTTTTTTTTTTCTCCAG	55
>hsa-miR-2276-3p MIMAT0011775	UCUGCAAGUGUCAGAGGCGAGG	CGGTCTGCAAGTGTCAGAG	GTGCAGGGTCCGAGGTCAGAGCCACCTGGGCAATTTTTTTTTTTCCTCGC	56
>hsa-miR-758-5p MIMAT0022929	GAUGGUUGACCAGAGAGCACAC	CGGGATGGTTGACCAGAGA	GTGCAGGGTCCGAGGTCAGAGCCACCTGGGCAATTTTTTTTTTTGTGTGC	56
>hsa-miR-373-3p MIMAT0000726	GAAGUGCUUCGAUUUUGGGGUGU	CGGGAAGTGCTTCGATTTTG	GTGCAGGGTCCGAGGTCAGAGCCACCTGGGCAATTTTTTTTTTTACACCC	56
>hsa-miR-21-5p MIMAT0000076	UAGCUUAUCAGACUGAUGUUGA	TTCGGTAGCTTATCAGACTGA	GTGCAGGGTCCGAGGTCAGAGCCACCTGGGCAATTTTTTTTTTTCAACAT	56
>hsa-miR-4689 MIMAT0019778	UUGAGGAGACAUGGUGGGGGCC	CGGTTGAGGAGACATGGTG	GTGCAGGGTCCGAGGTCAGAGCCACCTGGGCAATTTTTTTTTTTGGCCCC	56
>hsa-miR-106b-5p MIMAT0000680	UAAAGUGCUGACAGUGCAGAU	GTCGGTAAAGTGCTGACAGT	GTGCAGGGTCCGAGGTCAGAGCCACCTGGGCAATTTTTTTTTTTATCTGC	56
>hsa-miR-149-5p MIMAT0000450	UCUGGCUCCGUGUCUUCACUCCC	TGTCTGGCTCCGTGTCTTC	GTGCAGGGTCCGAGGTCAGAGCCACCTGGGCAATTTTTTTTTTTGGGAGT	56
>hsa-let-7b-5p MIMAT0000063	UGAGGUAGUAGGUUGUGUGGUU	TCGGTGAGGTAGTAGGTTGT	GTGCAGGGTCCGAGGTCAGAGCCACCTGGGCAATTTTTTTTTTTAACCAC	57
>hsa-miR-4692 MIMAT0019783	UCAGGCAGUGUGGGUAUCAGAU	TGGTCAGGCAGTGTGGGTA	GTGCAGGGTCCGAGGTCAGAGCCACCTGGGCAATTTTTTTTTTTATCTGA	57
>hsa-let-7d-3p MIMAT0004484	CUAUACGACCUGCUGCCUUUCU	TGGCTATACGACCTGCTGC	GTGCAGGGTCCGAGGTCAGAGCCACCTGGGCAATTTTTTTTTTTAGAAAG	57
>hsa-miR-877-5p MIMAT0004949	GUAGAGGAGAUGGCGCAGGG	TTCGGGTAGAGGAGATGGC	GTGCAGGGTCCGAGGTCAGAGCCACCTGGGCAATTTTTTTTTTTCCCTGC	57
>hsa-miR-15a-5p MIMAT0000068	UAGCAGCACAUAAUGGUUUGUG	TCGGTAGCAGCACATAATGG	GTGCAGGGTCCGAGGTCAGAGCCACCTGGGCAATTTTTTTTTTTCACAAA	57
>hsa-miR-105-5p MIMAT0000102	UCAAAUGCUCAGACUCCUGUGGU	TGGTCAAATGCTCAGACTCC	GTGCAGGGTCCGAGGTCAGAGCCACCTGGGCAATTTTTTTTTTTACCACA	57
>hsa-miR-3646 MIMAT0018065	AAAAUGAAAUGAGCCCAGCCCA	TTCGGAAAATGAAATGAGCCC	GTGCAGGGTCCGAGGTCAGAGCCACCTGGGCAATTTTTTTTTTTGGGCTG	57
>hsa-miR-181d-5p MIMAT0002821	AACAUUCAUUGUUGUCGGUGGGU	TCGGAACATTCATTGTTGTCG	GTGCAGGGTCCGAGGTCAGAGCCACCTGGGCAATTTTTTTTTTTACCCAC	58
>hsa-miR-34b-3p MIMAT0004676	CAAUCACUAACUCCACUGCCAU	TCGGCAATCACTAACTCCAC	GTGCAGGGTCCGAGGTCAGAGCCACCTGGGCAATTTTTTTTTTTATGGCA	58
>hsa-miR-455-5p MIMAT0003150	UAUGUGCCUUUGGACUACAUCG	TCGGTATGTGCCTTTGGACT	GTGCAGGGTCCGAGGTCAGAGCCACCTGGGCAATTTTTTTTTTTCGATGT	58
>hsa-miR-378a-5p MIMAT0000731	CUCCUGACUCCAGGUCCUGUGU	GGCTCCTGACTCCAGGTC	GTGCAGGGTCCGAGGTCAGAGCCACCTGGGCAATTTTTTTTTTTACACAG	58
>hsa-miR-196a-5p MIMAT0000226	UAGGUAGUUUCAUGUUGUUGGG	GTCGGTAGGTAGTTTCATGTT	GTGCAGGGTCCGAGGTCAGAGCCACCTGGGCAATTTTTTTTTTTCCCAAC	58
>hsa-miR-127-3p MIMAT0000446	UCGGAUCCGUCUGAGCUUGGCU	GGTCGGATCCGTCTGAGC	GTGCAGGGTCCGAGGTCAGAGCCACCTGGGCAATTTTTTTTTTTAGCCAA	58
>hsa-miR-590-5p MIMAT0003258	GAGCUUAUUCAUAAAAGUGCAG	TGTCGGGAGCTTATTCATAAAA	GTGCAGGGTCCGAGGTCAGAGCCACCTGGGCAATTTTTTTTTTTCTGCAC	58
>hsa-miR-320d MIMAT0006764	AAAAGCUGGGUUGAGAGGA	GGTCGGAAAAGCTGGGTTG	GTGCAGGGTCCGAGGTCAGAGCCACCTGGGCAATTTTTTTTTTTCCTCTC	59
>hsa-miR-140-3p MIMAT0004597	UACCACAGGGUAGAACCACGG	TTCGGTACCACAGGGTAGAA	GTGCAGGGTCCGAGGTCAGAGCCACCTGGGCAATTTTTTTTTTTCCGTGG	59
>hsa-miR-769-5p MIMAT0003886	UGAGACCUCUGGGUUCUGAGCU	TGGTGAGACCTCTGGGTTC	GTGCAGGGTCCGAGGTCAGAGCCACCTGGGCAATTTTTTTTTTTAGCTCA	59
>hsa-miR-330-5p MIMAT0004693	UCUCUGGGCCUGUGUCUUAGGC	GGTCTCTGGGCCTGTGTC	GTGCAGGGTCCGAGGTCAGAGCCACCTGGGCAATTTTTTTTTTTGCCTAA	59
>hsa-miR-1909-5p MIMAT0007882	UGAGUGCCGGUGCCUGCCCUG	TGGTGAGTGCCGGTGCCT	GTGCAGGGTCCGAGGTCAGAGCCACCTGGGCAATTTTTTTTTTTCAGGGC	59
>hsa-miR-548c-3p MIMAT0003285	CAAAAAUCUCAAUUACUUUUGC	TGTCGGCAAAAATCTCAATTAC	GTGCAGGGTCCGAGGTCAGAGCCACCTGGGCAATTTTTTTTTTTGCAAAA	59
>hsa-miR-1193 MIMAT0015049	GGGAUGGUAGACCGGUGACGUGC	GGGGATGGTAGACCGGTG	GTGCAGGGTCCGAGGTCAGAGCCACCTGGGCAATTTTTTTTTTTGCACGT	59
>hsa-miR-214-5p MIMAT0004564	UGCCUGUCUACACUUGCUGUGC	CGGTGCCTGTCTACACTTG	GTGCAGGGTCCGAGGTCAGAGCCACCTGGGCAATTTTTTTTTTTGCACAG	60
>hsa-miR-208a-3p MIMAT0000241	AUAAGACGAGCAAAAAGCUUGU	GTCGGATAAGACGAGCAAAAA	GTGCAGGGTCCGAGGTCAGAGCCACCTGGGCAATTTTTTTTTTTACAAGC	60
>hsa-miR-29c-3p MIMAT0000681	UAGCACCAUUUGAAAUCGGUUA	GTCGGTAGCACCATTTGAAAT	GTGCAGGGTCCGAGGTCAGAGCCACCTGGGCAATTTTTTTTTTTAACCGA	60
>hsa-miR-624-5p MIMAT0003293	UAGUACCAGUACCUUGUGUUCA	TCGGTAGTACCAGTACCTTG	GTGCAGGGTCCGAGGTCAGAGCCACCTGGGCAATTTTTTTTTTTGAACAC	60
>hsa-miR-660-5p MIMAT0003338	UACCCAUUGCAUAUCGGAGUUG	TCGGTACCCATTGCATATCG	GTGCAGGGTCCGAGGTCAGAGCCACCTGGGCAATTTTTTTTTTTCAACTC	60
>hsa-miR-30a-3p MIMAT0000088	CUUUCAGUCGGAUGUUUGCAGC	TCGGCTTTCAGTCGGATGTT	GTGCAGGGTCCGAGGTCAGAGCCACCTGGGCAATTTTTTTTTTTGCTGCA	60
>hsa-miR-26b-3p MIMAT0004500	CCUGUUCUCCAUUACUUGGCUC	TCGGCCTGTTCTCCATTACT	GTGCAGGGTCCGAGGTCAGAGCCACCTGGGCAATTTTTTTTTTTGAGCCA	60
>hsa-miR-331-5p MIMAT0004700	CUAGGUAUGGUCCCAGGGAUCC	TGGCTAGGTATGGTCCCAG	GTGCAGGGTCCGAGGTCAGAGCCACCTGGGCAATTTTTTTTTTTGGATCC	61
>hsa-miR-3651 MIMAT0018071	CAUAGCCCGGUCGCUGGUACAUGA	CATAGCCCGGTCGCTGGT	GTGCAGGGTCCGAGGTCAGAGCCACCTGGGCAATTTTTTTTTTTCATGTA	61
>hsa-miR-154-3p MIMAT0000453	AAUCAUACACGGUUGACCUAUU	TTCGGAATCATACACGGTTGA	GTGCAGGGTCCGAGGTCAGAGCCACCTGGGCAATTTTTTTTTTTAATAGG	61
>hsa-miR-218-2-3p MIMAT0004566	CAUGGUUCUGUCAAGCACCGCG	CGGCATGGTTCTGTCAAGC	GTGCAGGGTCCGAGGTCAGAGCCACCTGGGCAATTTTTTTTTTTCGCGGT	61

>hsa-miR-125b-5p MIMAT0000423	UCCCUGAGACCCUAACUUGUGA	TGGTCCCTGAGACCCTAAC	GTGCAGGGTCCGAGGTCAGAGCCACCTGGGCAATTTTTTTTTTTTCACAAG	61
>hsa-miR-1206 MIMAT0005870	UGUUCAUGUAGAUGUUUAAGC	TGTCGGTGTTCATGTAGATGT	GTGCAGGGTCCGAGGTCAGAGCCACCTGGGCAATTTTTTTTTTTTGCTTAA	61
>hsa-miR-504-5p MIMAT0002875	AGACCCUGGUCUGCACUCUAUC	GGAGACCCTGGTCTGCAC	GTGCAGGGTCCGAGGTCAGAGCCACCTGGGCAATTTTTTTTTTTTGATAGA	61
>hsa-miR-1307-3p MIMAT0005951	ACUCGGCGUGGCGUCGGUCGUG	GACTCGGCGTGGCGTCG	GTGCAGGGTCCGAGGTCAGAGCCACCTGGGCAATTTTTTTTTTTTCACGAC	62
>hsa-miR-302b-3p MIMAT0000715	UAAGUGCUUCCAUGUUUUAGUAG	TCGGTAAGTGCTTCCATGTTT	GTGCAGGGTCCGAGGTCAGAGCCACCTGGGCAATTTTTTTTTTTTCTACTA	62
>hsa-miR-221-3p MIMAT0000278	AGCUACAUUGUCUGCUGGGUUUC	TGGAGCTACATTGTCTGCTG	GTGCAGGGTCCGAGGTCAGAGCCACCTGGGCAATTTTTTTTTTTTGAAACC	62
>hsa-let-7f-5p MIMAT0000067	UGAGGUAGUAGAUUGUAUAGUU	TTCGGTGAGGTAGTAGATTGT	GTGCAGGGTCCGAGGTCAGAGCCACCTGGGCAATTTTTTTTTTTTAACTAT	62
>hsa-miR-216a-5p MIMAT0000273	UAAUCUCAGCUGGCAACUGUGA	TCGGTAATCTCAGCTGGCAA	GTGCAGGGTCCGAGGTCAGAGCCACCTGGGCAATTTTTTTTTTTTCACAGT	62
>hsa-miR-371b-3p MIMAT0019893	AAGUGCCCCCACAGUUUGAGUGC	GGAAGTGCCCCCACAGTTT	GTGCAGGGTCCGAGGTCAGAGCCACCTGGGCAATTTTTTTTTTTTGCACTC	62
>hsa-miR-142-3p MIMAT0000434	UGUAGUGUUUCCUACUUUAUGGA	TCGGTGTAGTGTTTCCTACTT	GTGCAGGGTCCGAGGTCAGAGCCACCTGGGCAATTTTTTTTTTTTCCATAA	62
>hsa-miR-424-5p MIMAT0001341	CAGCAGCAAUUCAUGUUUUGAA	TCGGCAGCAGCAATTCATGT	GTGCAGGGTCCGAGGTCAGAGCCACCTGGGCAATTTTTTTTTTTTCAAAAC	63
>hsa-miR-1181 MIMAT0005826	CCGUCGCCGCCACCCGAGCCG	GCCGTCGCCGCCACCC	GTGCAGGGTCCGAGGTCAGAGCCACCTGGGCAATTTTTTTTTTTTCGGCTC	63
>hsa-miR-661 MIMAT0003324	UGCCUGGGUCUCUGGCCUGCGCGU	TGCCTGGGTCTCTGGCCT	GTGCAGGGTCCGAGGTCAGAGCCACCTGGGCAATTTTTTTTTTTTACGCGC	63
>hsa-miR-363-3p MIMAT0000707	AAUUGCACGGUAUCCAUCUGUA	TCGGAATTGCACGGTATCCA	GTGCAGGGTCCGAGGTCAGAGCCACCTGGGCAATTTTTTTTTTTTACAGAT	63
>hsa-miR-30c-1-3p MIMAT0004674	CUGGGAGAGGGUUGUUUACUCC	TGGCTGGGAGAGGGTTGTT	GTGCAGGGTCCGAGGTCAGAGCCACCTGGGCAATTTTTTTTTTTTGGAGTA	63
>hsa-miR-93-3p MIMAT0004509	ACUGCUGAGCUAGCACUUCCCG	TGGACTGCTGAGCTAGCAC	GTGCAGGGTCCGAGGTCAGAGCCACCTGGGCAATTTTTTTTTTTTCGGGAA	63
>hsa-miR-193a-5p MIMAT0004614	UGGGUCUUUGCGGGCGAGAUGA	TGTGGGTCTTTGCGGGCG	GTGCAGGGTCCGAGGTCAGAGCCACCTGGGCAATTTTTTTTTTTTCATCTC	63
>hsa-miR-130b-5p MIMAT0004680	ACUCUUUCCCUGUUGCACUAC	TTCGGACTCTTTCCCTGTTG	GTGCAGGGTCCGAGGTCAGAGCCACCTGGGCAATTTTTTTTTTTTGTAGTG	64
>hsa-miR-3960 MIMAT0019337	GGCGGCGGCGGAGGCGGGGG	TGGGCGGCGGCGGAGG	GTGCAGGGTCCGAGGTCAGAGCCACCTGGGCAATTTTTTTTTTTTCCCCCG	64
>hsa-miR-1915-3p MIMAT0007892	CCCCAGGGCGACGCGGCGGG	GGCCCCAGGGCGACGC	GTGCAGGGTCCGAGGTCAGAGCCACCTGGGCAATTTTTTTTTTTTCCCGCC	64
>hsa-miR-371a-5p MIMAT0004687	ACUCAAACUGUGGGGGCACU	GTCGGACTCAAACTGTGGG	GTGCAGGGTCCGAGGTCAGAGCCACCTGGGCAATTTTTTTTTTTTAGTGCC	64
>hsa-miR-328-3p MIMAT0000752	CUGGCCCUCUCUGCCCUUCCGU	GCTGGCCCTCTCTGCCC	GTGCAGGGTCCGAGGTCAGAGCCACCTGGGCAATTTTTTTTTTTTACGGAA	64
>hsa-miR-30d-5p MIMAT0000245	UGUAAACAUCCCCGACUGGAAG	CGGTGTAAACATCCCCGAC	GTGCAGGGTCCGAGGTCAGAGCCACCTGGGCAATTTTTTTTTTTTCTTCCA	64
>hsa-miR-212-3p MIMAT0000269	UAACAGUCUCCAGUCACGGCC	TTCGGTAACAGTCTCCAGTC	GTGCAGGGTCCGAGGTCAGAGCCACCTGGGCAATTTTTTTTTTTTGGCCGT	64
>hsa-miR-1913 MIMAT0007888	UCUGCCCCCUCCGCUGCUGCCA	GTCTGCCCCCTCCGCTG	GTGCAGGGTCCGAGGTCAGAGCCACCTGGGCAATTTTTTTTTTTTGGCAGC	65
>hsa-miR-1587 MIMAT0019077	UUGGGCUGGGCUGGGUUGGG	CGGTTGGGCTGGGCTGG	GTGCAGGGTCCGAGGTCAGAGCCACCTGGGCAATTTTTTTTTTTTCCCAAC	65
>hsa-miR-548c-5p MIMAT0004806	AAAAGUAAUUGCGGUUUUUGCC	GTCGGAAAAGTAATTGCGGTT	GTGCAGGGTCCGAGGTCAGAGCCACCTGGGCAATTTTTTTTTTTTGGCAAA	65
>hsa-miR-1233-3p MIMAT0005588	UGAGCCCUGUCCUCCCGCAG	TCGGTGAGCCCTGTCCTC	GTGCAGGGTCCGAGGTCAGAGCCACCTGGGCAATTTTTTTTTTTTCTGCGG	65
>hsa-miR-4688 MIMAT0019777	UAGGGGCAGCAGAGGACCUGGG	GGTAGGGGCAGCAGAGGA	GTGCAGGGTCCGAGGTCAGAGCCACCTGGGCAATTTTTTTTTTTTCCCAGG	65
>hsa-miR-200a-5p MIMAT0001620	CAUCUUACCGGACAGUGCUGGA	CGGCATCTTACCGGACAGT	GTGCAGGGTCCGAGGTCAGAGCCACCTGGGCAATTTTTTTTTTTTCCAGCA	65
>hsa-miR-629-3p MIMAT0003298	GUUCUCCCAACGUAAGCCCAGC	CGGGTTCTCCCAACGTAAG	GTGCAGGGTCCGAGGTCAGAGCCACCTGGGCAATTTTTTTTTTTTGCTGGG	65
>hsa-miR-378e MIMAT0018927	ACUGGACUUGGAGUCAGGA	TGTCGGACTGGACTTGGAG	GTGCAGGGTCCGAGGTCAGAGCCACCTGGGCAATTTTTTTTTTTTCCTGAC	66
>hsa-miR-187-3p MIMAT0000262	UCGUGUCUUGUGUUGCAGCCGG	CGGTCGTGTCTTGTGTTGC	GTGCAGGGTCCGAGGTCAGAGCCACCTGGGCAATTTTTTTTTTTTCCGGCT	66
>hsa-miR-4289 MIMAT0016920	GCAUUGUGCAGGGCUAUCA	GTCGGGCATTGTGCAGGG	GTGCAGGGTCCGAGGTCAGAGCCACCTGGGCAATTTTTTTTTTTTGATAGC	66
>hsa-miR-500a-5p MIMAT0004773	UAAUCCUUGCUACCUGGGUGAGA	TGGTAATCCTTGCTACCTGG	GTGCAGGGTCCGAGGTCAGAGCCACCTGGGCAATTTTTTTTTTTTCTCACC	66
>hsa-miR-24-3p MIMAT0000080	UGGCUCAGUUCAGCAGGAACAG	TGGTGGCTCAGTTCAGCAG	GTGCAGGGTCCGAGGTCAGAGCCACCTGGGCAATTTTTTTTTTTTCTGTTC	66
>hsa-miR-32-5p MIMAT0000090	UAUUGCACAUUACUAAGUUGCA	TGTCGGTATTGCACATTACTAA	GTGCAGGGTCCGAGGTCAGAGCCACCTGGGCAATTTTTTTTTTTTGCAACT	66
>hsa-let-7d-5p MIMAT0000065	AGAGGUAGUAGGUUGCAUAGUU	CGGAGAGGTAGTAGGTTGC	GTGCAGGGTCCGAGGTCAGAGCCACCTGGGCAATTTTTTTTTTTTAACTAT	66
>hsa-miR-4786-5p MIMAT0019954	UGAGACCAGGACUGGAUGCACC	TGGTGAGACCAGGACTGGA	GTGCAGGGTCCGAGGTCAGAGCCACCTGGGCAATTTTTTTTTTTTGGTGCA	67
>hsa-miR-1207-5p MIMAT0005871	UGGCAGGGAGGCUGGGAGGGG	GGTGGCAGGGAGGCTGG	GTGCAGGGTCCGAGGTCAGAGCCACCTGGGCAATTTTTTTTTTTTCCCCTC	67
>hsa-miR-29b-3p MIMAT0000100	UAGCACCAUUUGAAAUCAGUGUU	TCGGTAGCACCATTTGAAATC	GTGCAGGGTCCGAGGTCAGAGCCACCTGGGCAATTTTTTTTTTTTAACACT	67
>hsa-miR-4306 MIMAT0016858	UGGAGAGAAAGGCAGUA	TCGGTCGGTGGAGAGAAAG	GTGCAGGGTCCGAGGTCAGAGCCACCTGGGCAATTTTTTTTTTTTACTGCC	67
>hsa-miR-301b-3p MIMAT0004958	CAGUGCAAUGAUAUUGUCAAAGC	TCGGCAGTGCAATGATATTGT	GTGCAGGGTCCGAGGTCAGAGCCACCTGGGCAATTTTTTTTTTTTGCTTTG	67

>hsa-miR-3185 MIMAT0015065	AGAAGAAGGCGGUCGGUCUGCGG	TGAGAAGAAGGCGGTCGGT	GTGCAGGGTCCGAGGTCAGAGCCACCTGGGCAATTTTTTTTTTTCCGCAG	67
>hsa-miR-136-3p MIMAT0004606	CAUCAUCGUCUCAAAUGAGUCU	TTCGGCATCATCGTCTCAAAT	GTGCAGGGTCCGAGGTCAGAGCCACCTGGGCAATTTTTTTTTTTAGACTC	67
>hsa-miR-495-3p MIMAT0002817	AAACAAACAUGGUGCACUUCUU	TTCGGAAACAAACATGGTGCA	GTGCAGGGTCCGAGGTCAGAGCCACCTGGGCAATTTTTTTTTTTAAGAAG	68
>hsa-miR-34b-5p MIMAT0000685	UAGGCAGUGUCAUUAGCUGAUUG	TGGTAGGCAGTGTCATTAGC	GTGCAGGGTCCGAGGTCAGAGCCACCTGGGCAATTTTTTTTTTTCAATCA	68
>hsa-miR-92a-1-5p MIMAT0004507	AGGUUGGGAUCGGUUGCAAUGCU	TGAGGTTGGGATCGGTTGC	GTGCAGGGTCCGAGGTCAGAGCCACCTGGGCAATTTTTTTTTTTAGCATT	68
>hsa-miR-4302 MIMAT0016855	CCAGUGUGGCUCAGCGAG	TGTCGGCCAGTGTGGCTC	GTGCAGGGTCCGAGGTCAGAGCCACCTGGGCAATTTTTTTTTTTCTCGCT	68
>hsa-miR-4291 MIMAT0016922	UUCAGCAGGAACAGCU	TTCGGTCGGTTCAGCAGGA	GTGCAGGGTCCGAGGTCAGAGCCACCTGGGCAATTTTTTTTTTTAGCTGT	68
>hsa-miR-486-5p MIMAT0002177	UCCUGUACUGAGCUGCCCCGAG	TGGTCCTGTACTGAGCTGC	GTGCAGGGTCCGAGGTCAGAGCCACCTGGGCAATTTTTTTTTTTCTCGGG	68
>hsa-miR-28-5p MIMAT0000085	AAGGAGCUCACAGUCUAUUGAG	CGGAAGGAGCTCACAGTCT	GTGCAGGGTCCGAGGTCAGAGCCACCTGGGCAATTTTTTTTTTTCTCAAT	68
>hsa-miR-423-3p MIMAT0001340	AGCUCGGUCUGAGGCCCCUCAGU	TAGCTCGGTCTGAGGCCC	GTGCAGGGTCCGAGGTCAGAGCCACCTGGGCAATTTTTTTTTTTACTGAG	69
>hsa-let-7a-5p MIMAT0000062	UGAGGUAGUAGGUUGUAUAGUU	TCGGTGAGGTAGTAGGTTGT	GTGCAGGGTCCGAGGTCAGAGCCACCTGGGCAATTTTTTTTTTTAACTAT	69
>hsa-miR-514a-3p MIMAT0002883	AUUGACACUUCUGUGAGUAGA	GTCGGATTGACACTTCTGTG	GTGCAGGGTCCGAGGTCAGAGCCACCTGGGCAATTTTTTTTTTTCTACTC	69
>hsa-miR-370-3p MIMAT0000722	GCCUGCUGGGGUGGAACCUGGU	TGGCCTGCTGGGGTGGAA	GTGCAGGGTCCGAGGTCAGAGCCACCTGGGCAATTTTTTTTTTTACCAGG	69
>hsa-miR-132-3p MIMAT0000426	UAACAGUCUACAGCCAUGGUCG	TCGGTAACAGTCTACAGCCA	GTGCAGGGTCCGAGGTCAGAGCCACCTGGGCAATTTTTTTTTTTCGACCA	69
>hsa-miR-21-3p MIMAT0004494	CAACACCAGUCGAUGGGCUGU	TCGGCAACACCAGTCGATG	GTGCAGGGTCCGAGGTCAGAGCCACCTGGGCAATTTTTTTTTTTACAGCC	69
>hsa-miR-154-5p MIMAT0000452	UAGGUUAUCCGUGUUGCCUUCG	TCGGTAGGTTATCCGTGTTG	GTGCAGGGTCCGAGGTCAGAGCCACCTGGGCAATTTTTTTTTTTCGAAGG	69
>hsa-miR-346 MIMAT0000773	UGUCUGCCCGCAUGCCUGCCUCU	GTGTCTGCCCGCATGCCT	GTGCAGGGTCCGAGGTCAGAGCCACCTGGGCAATTTTTTTTTTTAGAGGC	70
>hsa-miR-103a-3p MIMAT0000101	AGCAGCAUUGUACAGGGCUAUGA	GGAGCAGCATTGTACAGGG	GTGCAGGGTCCGAGGTCAGAGCCACCTGGGCAATTTTTTTTTTTCATAGC	70
>cel-miR-54-5p MIMAT0020773	AGGAUAUGAGACGACGAGAACA	TCGGAGGATATGAGACGACG	GTGCAGGGTCCGAGGTCAGAGCCACCTGGGCAATTTTTTTTTTTGTTCTC	
Universal reverse primer	CAGTGCAGGGTCCGAGGT			
Universal Taqman probe	56-FAM/CAGAGCCAC/ZEN/CTGGGCAATTT/3IABkFQ			
